# Progress in Nanofluid Technology: From Conventional to Green Nanofluids for Biomedical, Heat Transfer, and Machining Applications

**DOI:** 10.3390/nano15161242

**Published:** 2025-08-13

**Authors:** Beatriz D. Cardoso, Andrews Souza, Glauco Nobrega, Inês S. Afonso, Lucas B. Neves, Carlos Faria, João Ribeiro, Rui A. Lima

**Affiliations:** 1 Mechanical Engineering and Resource Sustainability Center (MEtRICs), Mechanical Engineering Department, University of Minho, Campus de Azurém, 4800-058 Guimarães, Portugal; andrewsv81@gmail.com (A.S.); glaucotvn@hotmail.com (G.N.); inesafonso@ipb.pt (I.S.A.); neves.lucas17@gmail.com (L.B.N.); carlosajlfaria2@gmail.com (C.F.); 2 Centro de Investigação de Montanha (CIMO), Instituto Politécnico de Bragança, Campus de Santa Apolónia, 5300-253 Bragança, Portugal; jribeiro@ipb.pt; 3 CMEMS-UMinho, University of Minho, Campus de Azurém, 4800-058 Guimarães, Portugal; 4 International Iberian Nanotechnology Laboratory (INL), 4715-330 Braga, Portugal; 5 Associate Laboratory in Chemical Engineering (ALiCE), University of Porto, 4200-465 Porto, Portugal; 6 Transport Phenomena Research Center (CEFT), Faculdade de Engenharia da Universidade do Porto (FEUP), Rua Roberto Frias, 4200-465 Porto, Portugal

**Keywords:** nanofluids, heat transfer, nanoparticles, thermal conductivity, green synthesis, applications

## Abstract

Nanofluids (NFs), consisting of nanoparticles (NPs) suspended in base fluids, have attracted growing interest due to their superior physicochemical properties and multifunctional potential. In this review, conventional and green NF technology aspects, including synthesis routes, formulation, and applications, are discussed. Conventional NFs, involving NPs synthesized using physical and chemical approaches, have improved NP morphology control but are likely to cause environmental and safety concerns. In contrast, green NFs that are plant extract, microorganism, and biogenic waste-based represent a sustainable and biocompatible alternative. The effect of key parameters (e.g., NP size, shape, concentration, dispersion stability, and base fluid properties) on the performance of NFs is critically examined. The review also covers potential applications: in biomedical engineering (e.g., drug delivery, imaging, theranostics, and antimicrobial therapies), in heat transfer (e.g., solar collectors, cooling electronics, nuclear reactors), and precision machining (e.g., lubricants and coolants). Comparative insights regarding green versus conventionally prepared NFs are provided concerning their toxicity, environmental impact, scalability, and functional performance across various applications. Overall, this review highlights the new promise of both green and conventional NFs and provides key opportunities and challenges to guide future developments in this field.

## 1. Introduction

**Figure 1:**
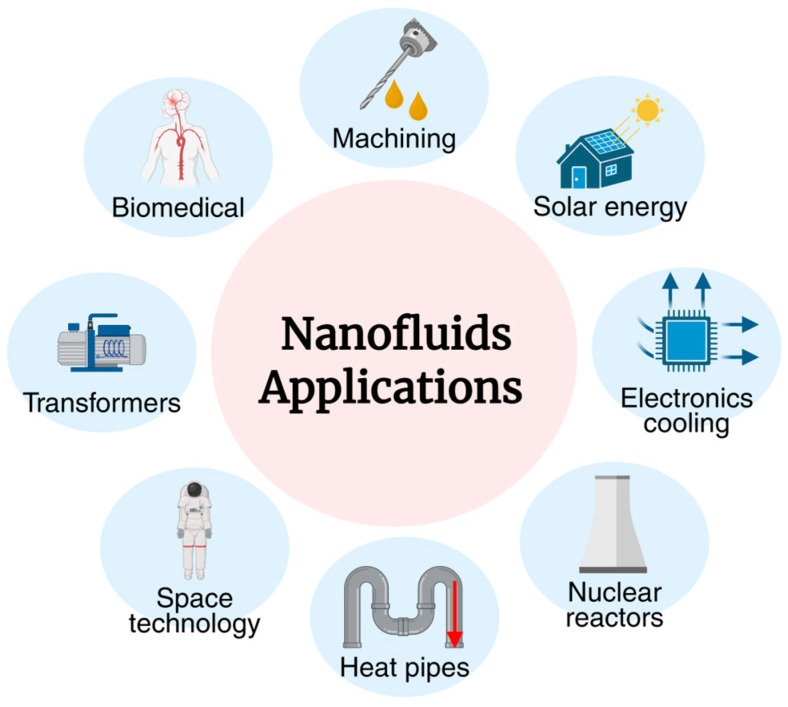
Overview of engineering applications of NFs, including both conventional and green approaches.

Nanofluids (NFs) are engineered colloidal suspensions of nanoparticles (NPs), in the range of 1–100 nm, dispersed in conventional base fluids such as water, oil, or ethylene glycol. Incorporating nanomaterials (for instance, metals, metal oxides, carbides, and carbon particles) into these fluids significantly enhances their thermophysical properties, especially thermal conductivity, viscosity, and specific heat [1,2]. While Choi [3,4] was the first to propose the use of NPs for enhancing heat transfer, the concept of suspending solid particles in liquids to improve thermal performance dates back to the early 20th century [5]. One of the earliest achievements in the field was in 1997 when Eastman et al. [6] enhanced the thermal conductivity of water and oil using NPs. Now, NFs continue to be of high research interest, with the greatest potential advances resulting from inter-disciplinary research collaborations (Figure 1).

The most studied and applied application of NFs still remains in thermal management systems, including heat exchangers, electronic cooling systems, solar collectors, and internal combustion engines [7,8]. Their enhanced ability to dissipate heat makes them a favorable option for high-performance compact systems in energy and industrial sectors [9]. Such systems include engine radiators, solar collectors, heat exchangers, electronics cooling, heat pipes, and even nuclear reactors. Their advantages are related directly to the thermophysical performance of the fluid at high thermal loading; thus, optimization of stability and thermal conductivity is needed [10,11,12].

In biomedical engineering, NFs show promise in a range of innovative technologies. Applications include magnetic hyperthermia for cancer therapy, targeted drug delivery, bioimaging, and theranostics (combined use of diagnostic and therapeutic agents) [13,14,15]. The biocompatibility and tunable surface properties of NFs, especially those synthesized via green methods, allow for better control of cellular uptake, circulation time, and therapeutic effectiveness [16].

Besides traditional thermal applications, NFs are being increasingly used in precision machining (turning, drilling, milling, and grinding) as nano-lubricants and coolants [17,18]. High thermal conductivity, improved wettability, and good tribological properties of NFs are accountable for reducing tool wear, cutting forces, surface roughness, and improving tool life [19]. These benefits are more recognizable when blended with environmentally friendly methods like minimum quantity lubrication (MQL) [20].

To meet the growing demand for safer, more sustainable solutions, green NFs have gained attention [21]. These formulations use NPs synthesized through environmentally friendly methods, often using plant extracts or microorganisms, offering reduced toxicity and improved biocompatibility. Despite having broad potential, NFs performance is very dependent on the type of NP, size, geometry, concentration, and quality of dispersion [22,23]. Furthermore, agglomeration, long-term stability, and compatibility with the fluid are concerns that need to be addressed to ensure stable operation in systems. 

This review aims to provide a topical overview of both conventional and green NFs with emphasis on synthesis methods, thermophysical properties, and economic, environmental, and commercialization issues of NFs. Special emphasis is laid on their applications in three lead fields: biomedical systems, heat transfer technologies, and machining operations.

## 2. Overview of Conventional and Green Nanofluids Production

### 2.1. Conventional Synthesis of Nanoparticles

**Figure 2:**
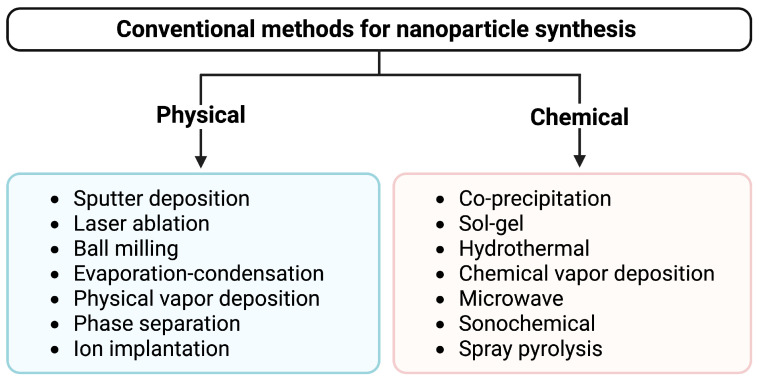
Summary of physical and chemical methods used in conventional nanoparticle synthesis approaches.

Conventional methods of synthesizing NPs include chemical and physical methods Figure 2 summarizes some of the most employed approaches within both categories. These techniques have been used for decades as they offer precise control of size, shape, and purity of the NPs [24,25,26]. Conventional synthesis uses either top-down or bottom-up strategies. In top-down methods, bulk materials are broken down into NPs using tools, for instance, ball milling or lasers [27,28]. Bottom-up methods build NPs from atoms or molecules using techniques such as vapor deposition or sol-gel processes [29,30,31]. Chemical synthesis employs strong chemical reducing agents such as sodium borohydride or hydrazine to transform metal salts into NPs of metals [32,33]. Agents such as surfactants or polymers are usually used to prevent the NPs from aggregating. Chemical synthesis allows precise control of NPs characteristics by adjusting the conditions of the reaction [24]. Furthermore, these methods are well-suited for large-scale production, making them ideal for industrial applications where high volumes of NPs are required [34].

Physical synthesis methods are valued for their ability to produce NPs with precise control over size and shape and with high purity, as they do not require chemical reagents that can introduce impurities [35,36]. These techniques are compatible with a wide range of materials. However, they often require high energy input, operate under extreme conditions, and are generally less environmentally friendly and more costly compared to other approaches [37,38]. Generally, chemical approaches may use toxic chemicals and produce toxic by-products, while physical methods involve costly equipment and vast amounts of energy, and thus are less eco-friendly and costly to upscale. 

### 2.2. Green Synthesis of Nanoparticles

#### 2.2.1. Principles of Green Synthesis

In recent years, the growing emphasis on sustainability and environmentally friendly practices has driven significant progress in NP synthesis. Among these advancements, green synthesis has emerged as a promising and eco-conscious approach for producing nanoscale materials while minimizing ecological and health-related risks [39,40].

It employs eco-friendly reagents, renewable resources, and energy-efficient methods, while minimizing the generation of hazardous by-products [34,41,42]. This approach follows sustainability principles, including the efficient use of resources, waste reduction, and the protection of future generations [43,44]. Moreover, it emphasizes the use of renewable and sustainable resources (such as plant extracts, microorganisms, and agricultural waste) as raw materials for synthesis, given their abundance and minimal reliance on finite natural reserves [45,46]. In contrast to traditional chemical synthesis, which often relies on hazardous reducing agents, green methods use safer solvents (e.g., water or ethanol) and natural reducing agents to produce and stabilize NPs [47,48,49]. For instance, polyphenol-rich plant extracts or microbial cells can reduce metal ions while simultaneously preventing aggregation, avoiding the production of harmful by-products [50,51]. These strategies align with circular economy principles and offer a sustainable route to nanomaterials, particularly by using agricultural residues such as rice husks or fruit peels [45,52]. Microbial agents like bacteria and fungi also play a critical role due to their inherent capacity to reduce metal ions and facilitate NP formation [53,54].

**Figure 3:**
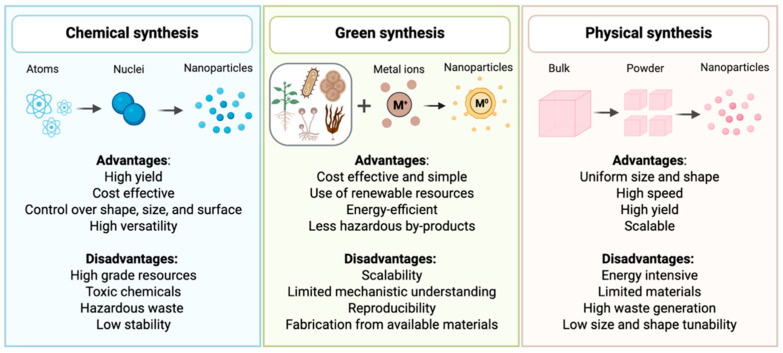
Main advantages and disadvantages of the NP synthesis using chemical, green, and physical methods. Reproduced from [42].

Figure 3 presents the main advantages and disadvantages of chemical, physical, and green methods. This comparison underscores the growing preference for green synthesis as a safer and more sustainable alternative to conventional techniques.

#### 2.2.2. The Role of Plants and Microorganisms in Nanoparticle Synthesis

Plants play a central role in green NPs synthesis due to their rich content of naturally occurring bioactive compounds. Extracts from various plant parts (such as leaves, stems, roots, and flowers) contain molecules like polyphenols, flavonoids, terpenoids, and sugars, which can act as effective reducing and stabilizing agents during NPs formation [55,56]. The bioactive molecules donate electrons to the metal ions (e.g., silver or gold ions), where they are reduced to their metallic zero-valent form. This means that the metal ions lose their charge to become simple metal atoms that start to clump together. Once the NPs are established, biomolecules bind onto their surface to act as a capping layer to avoid the particles from clumping together [57,58,59]. Similarly to plants, algae, which are water-dwelling organisms, also play a role in NPs synthesis [60]. Algae possess natural reducers like chlorophyll, carotenoids, and antioxidants, which are bioactive compounds present within them [61,62]. If metal ions are introduced into an algal extract, these compounds donate electrons to reduce ions to metal atoms, thereby initiating the NPs formation, and provide a capping effect [63,64,65,66]. 

Yeast, a fungus that is extensively used in food and beverage production, is another effective biological agent for green synthesis [67]. Yeast cells also facilitate the production of NPs intracellularly as well as extracellularly [68]. Intracellularly, metal ions are reduced to NPs by the action of enzymes, while extracellularly, secreted enzymes perform the same function in the external solution [68,69,70]. Yeast secretes its natural compounds like nitrate reductase and other enzymes that act as biological “helpers” to reduce metal ions to a metallic state [71,72]. Biomolecules such as proteins, sugars, and other yeast organic molecules not only catalyze the reduction process but also act as natural stabilizers (or capping agents) by surrounding newly formed NPs [72]. 

Bacteria also enable NPs production through their metabolic processes and the manufacture of enzymes. The synthesis can occur inside bacterial cells or outside by enzymes that are excreted to the external environment [73,74]. Bacteria produce specific enzymes such as nitrate reductase, hydrogenase, or oxidoreductases [42]. These enzymes catalyze electron transfer to metal ions, reducing them (or “activating” them) so that they grow together to create NPs. Bacteria can also excrete substances called extracellular polymeric substances (EPS) that enable “capping” and stabilization of NPs so that they do not agglomerate [75]. Bacterial synthesis has been found to be very efficient, cost-effective, and environmentally friendly. 

Finally, fungi such as molds and mushrooms are also a significant source for green NPs synthesis [76]. Fungi react with metal ions dissolved in solution. Extracellular enzymes such as laccase, reductase, and peroxidase are utilized by fungi to reduce metal ions to NPs [77,78]. Intracellular synthesis within the fungal cell or extracellular synthesis outside the cell, where the formed NPs are stabilized by fungal secreted proteins and polysaccharides, depending on the case [77,78,79,80]. 

### 2.3. Conventional and Green Nanofluid Formulation Techniques

NFs are usually produced using one of two main procedures: the one-step method or the double-step method [3,5,81]. The one-step approach combines NPs synthesis and dispersion into the base fluid in a single process, which generally results in improved stability. NPs in this method are commonly produced via physical vapor deposition (e.g., carbon nanotubes) or chemical liquid-phase techniques (e.g., copper (Cu) NFs) [81,82,83]. In contrast, the double-step method involves the initial synthesis of NPs, followed by their dispersion into the base fluid. This approach uses various synthesis techniques, including inert gas condensation (used for aluminum (Al), Cu, molybdenum (Mo), platinum (Pt), titanium (Ti), and iron oxide NPs) [84], mechanical alloying (trinickel disulfide (Ni_3_S_2_), Mg_2_Ti_4_, cobalt ferrite (CoFe_2_O_4_)) [85,86,87], chemical vapor deposition (boron nitride (BN) nanotubes, carbon nanotubes, magnetite (Fe_3_O_4_)) [88,89,90], or chemical deposition (PbS) [91]. After undergoing separation and drying, the NPs are dispersed into the base fluid [81,92]. To address the increased risk of agglomeration during this stage, methods such as ultrasonic agitation and the use of surfactants are applied to promote uniform dispersion and improve thermal performance [81].

**Figure 4:**
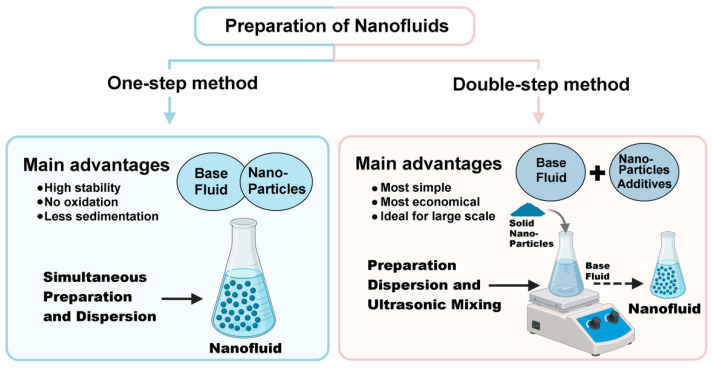
Preparation of NFs through the one-step and double-step methods.

In summary, one-step method favors enhanced compatibility and performance through superior stability and dispersion, but face limitations in scalability and cost. Double-step method is more industrially viable and economical but requires careful optimization to maintain performance and compatibility (see Figure 4). The integration of sustainable synthesis techniques further improves environmental compatibility, aligning with modern industrial and regulatory demands [93,94,95].

## 3. Factors Affecting the Physicochemical Properties of Nanofluids

The properties of NPs offer significant performance advantages over base fluids, making them promising candidates for various engineering and industrial applications [96]. Given this versatility and the complexity of their formulation, the selection and design of NFs must be made to the specific requirements of each application. The overall properties of NFs can be influenced by various factors, as preparation parameters often vary significantly. Key characteristics of the NPs (such as their size, shape, concentration, and stability within the fluid), along with the properties of the base fluid and the degree of dispersion, can all directly or indirectly affect overall performance.

### 3.1. Size of Nanoparticles

NPs’ size is a key parameter that directly affects biological interactions, colloidal stability, and functional performance in biomedical applications. Small NPs possess a higher surface area-to-volume ratio, thus enhancing reactivity, solubility, and ease of functionalization, all relevant for drug delivery, imaging, and biosensing [97,98]. However, the optimum size varies according to the application. NPs with sizes less than 10 nm can permeate through membranes or be cleared quickly by renal excretion, while NPs of between 20 and 150 nm take advantage of the enhanced permeability and retention (EPR) effect for selective drug delivery [99,100]. Endocytosis selectively takes up particles of 10–100 nm size, though precise uptake and intracellular fate are very much size-dependent [101,102].

In therapeutic and imaging applications, size decides the physicochemical properties of NPs such as optical, magnetic, and plasmonic features. Quantum dots (1–10 nm) exhibit size-tunable fluorescence [103], Au and Ag NPs (10–100 nm) exhibit plasmonic functionalities for SERS and photothermal therapy [104,105]. Iron oxide NPs (5–50 nm) exhibit size-tunable magnetic functionalities for MRI and magnetothermal therapy [106]. Interestingly, large NPs can generate more signal but present a lower stability, biodistribution, and clearance [107,108]. Thus, strict size control is required to obtain performance vs. biocompatibility.

The size of NPs also controls thermal conductivity. Smaller NPs (<100 nm) generally enhance heat transfer by enhancing surface contact, Brownian motion, and the development of interfacial layers [109,110,111,112,113,114]. Ambreen et al. [115] showed that decreasing aluminum oxide (Al_2_O_3_) and titanium dioxide (TiO_2_) NP sizes (20–200 nm) enhanced heat transfer, but the improvement leveled off for sizes below 40 nm. Qin et al. [116] also reported increased conductivity with Al_2_O_3_ NPs as particle size decreased from 100 nm to 5 nm, specifically for higher concentrations. Similarly, Hemmat Esfe et al. [117] reported that smaller Fe NPs increased thermal conductivity but larger sizes increased viscosity, both impacting flow and energy efficiency.

Overall, the gains in thermal performance and biomedical activity decrease when smaller NPs are used below 40–50 nm, and stability problems can occur. NF performance is optimized through an equilibrium among size and other parameters like concentration, form, and base fluid properties.

### 3.2. Shape of Nanoparticles

NPs shape plays a significant role in biomedical as well as in thermal applications. Cellular uptake, drug delivery, and therapeutic outcome are influenced by NP geometry in biological systems. Rods and triangles are examples of anisotropic geometries that generally internalize better inside cells than spherical NPs due to heterogeneity in membrane wrapping kinetics and protein affinity behavior [118,119]. For example, Au nanorods are more efficient than spheres in photothermal therapy due to greater infrared absorption. However, sharp-edged geometry can damage vascular tissues, and hence careful morphological design is the emphasis [119]. Shape also controls key optical properties that are of interest for biosensing and imaging; triangular-shaped NPs, possessing sharp tips, give rise to more intense localized field enhancements than rods and spheres and therefore are especially suited to advanced diagnostic applications [120,121]. However, shape effects tend to co-vary with size and surface chemistry, so isolating their separate contributions is challenging [122].

In thermal systems, most experimental and theoretical studies have demonstrated that the NP shape has a significant influence on modifying the thermal conductivity of NFs. High-aspect-ratio morphologies such as rods, wires, nanotubes, and sheets always result in enhanced thermal conductivity. This is largely due to their increased surface area, which enhances particle–fluid interaction, as well as their ability to form continuous conducting networks that reduce interfacial thermal resistance [123,124,125,126]. Kim et al. [127] investigated Al boehmite NPs with brick, platelet, and blade shapes and achieved thermal conductivity improvements of 28%, 23%, and 16%, respectively. The brick shape yielded better suspension stability, which led to higher thermal enhancement. Similarly, Maheshwary et al. [128] evaluated TiO_2_ NFs at a concentration of 2.5 wt.% and observed that cubic NPs yielded the highest thermal gains, followed by rods and spheres. This was attributed to the larger surface area of cubic NPs, enabling them to couple thermally with the base fluid in a better way. In addition to this, Cui et al. [129] employed both experimental methods and AI modeling to test TiO_2_ NPs with spherical, ellipsoidal, clubbed, and sheet-like morphologies. As per them, leaf-shaped (sheet-like) particles were found to improve thermal conductivity the most, especially at elevated temperatures (60 °C) and concentrations (4 vol.%). Together, these research works emphasize that maximizing NP shape is significant to increase NF thermal efficiency by facilitating better thermal routes.

### 3.3. Concentration of Nanoparticles

NP concentration is of the most critical concern in both thermal and biomedical use. Biomedical use is controlled by therapeutic efficacy, biodistribution, protein corona formation, cellular uptake, and safety profiles [130,131,132]. Too low concentrations result in reduced efficacy or negative biological response, whereas increased concentrations cause toxicity or NP agglomeration. For example, Ag NPs exhibit concentration-dependent activity against *Pseudomonas aeruginosa* biofilms: low concentrations (~2 µg/mL) will inhibit maturation but also trigger bacterial defense in the form of increased EPS production, whereas higher concentrations (12–18 µg/mL) are disruptive to biofilm morphology [133]. Similarly, Co NPs released from metal-on-metal implants are dose-dependently cytotoxic to macrophages at >10^12^ particles/mL, driven by NP uptake and intracellular corrosion to oxidative stress [134,135,136]. These findings highlight the need for precise and calibrated measurement of concentration in biological matrices, where dynamic behavior and agglomeration can frustrate control over dosing.

In thermal applications, NP concentration has a significant role to play in controlling the thermal conductivity of NF. Higher volumetric fractions have the effect of enhancing heat transfer through enhanced particle–fluid contact area and greater thermal bridging through controlled agglomeration [137,138,139]. Jana et al. [140] established a linear relationship between NP concentration and thermal conductivity in Cu–water NFs and stated that suspension stability is highly crucial; too high concentrations can lead to sedimentation, which reduces conductivity over time. Maheshwary et al. [128] studied TiO_2_ NFs (0.5–2.5 wt.%) and noted up to 54% enhanced conductivity. Statistical modeling showed concentration as the most significant parameter (69.23%), followed by particle size (24.85%) and shape (5.54%). Although they increase conductivity, high levels can also compromise the stability of the system.

### 3.4. Dispersion of Nanoparticles

The formation of agglomerates can have positive or negative effects, depending on the degree of control over the system. When well distributed, especially in formulations with small particles and adequate concentrations, these structures can create additional paths for heat conduction, contributing to increased thermal conductivity [140,141]. In systems with magnetic NPs, this effect can be manipulated more precisely through the application of external magnetic fields, which organize the clusters in a reversible and efficient way, favoring thermal performance [142,143]. Li et al. [144] reported a significant increase in the conductivity of iron nanofibers under magnetic fields applied in different directions. observed increased conductivity in iron-based nanofibers subjected to a magnetic field. 

### 3.5. Intrinsic Thermal Conductivity of Nanoparticles

Several kinds of NPs have been investigated for improving the thermal efficacy of NFs. Some studies indicate that the intrinsic thermal conductivity of NPs has a low impact, while others demonstrate a direct association between increased NP thermal conductivity and enhanced heat transfer in NFs [142]. Wang et al. [145] reported that Cu-based NFs demonstrated enhanced thermal conductivity compared to Al-based fluids, attributed to copper’s improved thermal characteristics. In contrast, Yoo et al. [146] observed greater conductivity increases with TiO_2_ NPs compared to Al_2_O_3_, despite TiO_2_ possessing lower intrinsic thermal conductivity. 

### 3.6. Properties of the Base Fluid

Thermal characteristics of NFs are highly influenced by the properties of the base fluid. In general, fluids that naturally possess high thermal conductivity exhibit smaller relative increases in the presence of NPs [142]. Interfacial layer formation by NPs and interactions among fluids, significant in heat conduction, are also dependent on these interactions [147,148,149]. Agarwal et al. [150] tested Al_2_O_3_-based NFs with distilled water and ethylene glycol (maximum of 2% concentration, 10–70 °C), reporting greater thermal gains using ethylene glycol (up to 31%), as a result of greater NP-fluid interaction. The same authors, in another experiment with CuO NPs, employed distilled water for the greatest thermal conductivity enhancement (40%), which was followed by ethylene glycol (27%) and motor oil (19%) [151]. These variations indicate the behavior of the interfacial layer to be a determining factor. Moreover, Sundar et al. [152] studied nanodiamond NFs in three ethylene glycol–water mixtures with different ratios (20:80, 40:60, 60:40) and observed that higher content of water (owing to its greater thermal conductivity and reduced viscosity) offered greater performance, up to 17.8% improvement at 60 °C and 1% volume of NP.

Generally, NF thermal conductivity depends on various interacting parameters: NP shape, size, type, concentration, base fluid characteristics, and suspension stability. The parameters need to be optimized for maximum efficiency in industrial and technological processes.

## 4. Biomedical Applications of Nanofluids

NFs have attracted increasing interest in biomedicine for applications such as cancer therapy, drug delivery, imaging, and diagnostics, largely due to their responsiveness to external stimuli, surface functionalization capabilities, and potential for targeted delivery. Among these, magnetic NPs (MNPs) and nanofibers (NFs) stand out for their superparamagnetic behavior, anisotropy, and high surface area, enabling multifunctional use in nanotheranostics (integrated diagnostic and therapeutic systems) [153,154,155,156,157,158]. However, the synthesis route of nanomaterials plays a crucial role in determining their biomedical suitability. The following subsections explore biomedical applications of NFs, categorized by their synthesis route (conventional versus green synthesis) to highlight how these differences impact their clinical potential.

### 4.1. Biomedical Applications of Conventional Nanofluids

#### 4.1.1. Targeted Drug Delivery and Cancer Therapy

Inorganic NPs have emerged as a promising strategy in cancer therapy due to their ability to enhance drug targeting, reduce systemic toxicity, and enable controlled release. Their nanoscale size facilitates circulation in the bloodstream and penetration through biological barriers, including the blood-brain barrier (BBB) [159]. Functionalization with ligands or antibodies further improves tumor-specific accumulation by prolonging circulation time and enhancing active targeting [160]. NPs leverage the EPR effect, wherein leaky tumor vasculature promotes preferential accumulation at the tumor site [161]. This property also enables synergistic therapeutic strategies such as magnetic hyperthermia and photothermal therapy (PTT) [162,163]. Magnetic hyperthermia uses superparamagnetic NPs under an alternating magnetic field (AMF) to induce localized heat, while PTT involves the conversion of near-infrared light into cytotoxic heat via photothermal agents. Both approaches exploit the thermal sensitivity of cancer cells, which can be selectively damaged by mild temperature elevations of 5–7 °C, sparing healthy tissues [164,165].

Several studies have demonstrated the versatility of NPs-based platforms for targeted therapy. Folate-functionalized Fe_3_O_4_@Au NPs coated with dextran and loaded with curcumin (Fe_3_O_4_@Au-DEX-CU-FA) have shown targeted efficacy against liver cancer cells [166]. These ~63 nm NPs provided pH-responsive curcumin release, high biocompatibility, and selective cytotoxicity toward SNU-449 cells, while sparing normal hepatocytes. Molecular docking supported their interaction with apoptotic proteins BCL-XL and BAK, and in vivo studies confirmed tumor reduction and immune modulation [166].

Another promising platform involved Fe_3_O_4_@SiO_2_/MIL-100(Fe), a hybrid nanocarrier combining magnetic targeting with a porous metal-organic framework [167]. This system achieved high ciprofloxacin (CIP) loading under acidic conditions (97.5% at pH 5), with pH-dependent drug release favorable for tumor environments. Selective cytotoxicity toward MCF-7 cells and reduced off-target effects underline its therapeutic relevance [167].

Fe_3_O_4_ –Ag hybrid NPs, synthesized via in situ reduction into core–shell and heteromer structures, further extended therapeutic functionality [168]. These hybrids maintained magnetic hyperthermia capabilities while benefiting from the cytotoxic effects of silver ions. In vitro assays confirmed high biocompatibility and enhanced cancer cell killing under magnetic fields. In vivo, these NPs significantly suppressed tumor growth in mice through synergistic heat and ion-mediated apoptosis, with minimal toxicity to healthy tissues. Furthermore, Au NPs have demonstrated significant potential in enhancing breast cancer therapy through photothermal conversion under visible light irradiation [169]. This photothermal effect led to substantial cancer cell membrane disruption and cytotoxicity, with a reported 60% reduction in MCF-7 cell viability under PTT alone. Moreover, the combination of Au NP-mediated PTT with doxorubicin (DOX) results in synergistic therapeutic outcomes. The combined treatment yielded greater reductions in cell viability compared to either therapy alone, attributed to hyperthermia-induced impairment of DNA repair mechanisms that enhance DOX activity [169].

Finally, amino acid-functionalized Fe_3_O_4_ NPs synthesized via coprecipitation showed strong potential for multimodal therapy [170]. Coatings with glycine, β-alanine, L-phenylalanine, or D-phenylglycine improved colloidal stability and biocompatibility. These NPs exhibited robust magnetic properties (65–70 emu/g), efficient hyperthermia performance (SAR up to 81 W/g), and antiplasmodial activity, suggesting utility in both cancer treatment and infectious disease management.

#### 4.1.2. Theranostics and Imaging

In addition to their therapeutic activity, MNPs act as non-invasive contrast agents for magnetic resonance imaging (MRI), facilitating integrated theranostic applications (therapy + diagnostic) [171]. FeO NPs are among the most extensively explored metal oxide NPs in theranostic applications, owing to their distinctive magnetic properties that support both diagnostic imaging and therapeutic interventions. 

**Figure 5:**
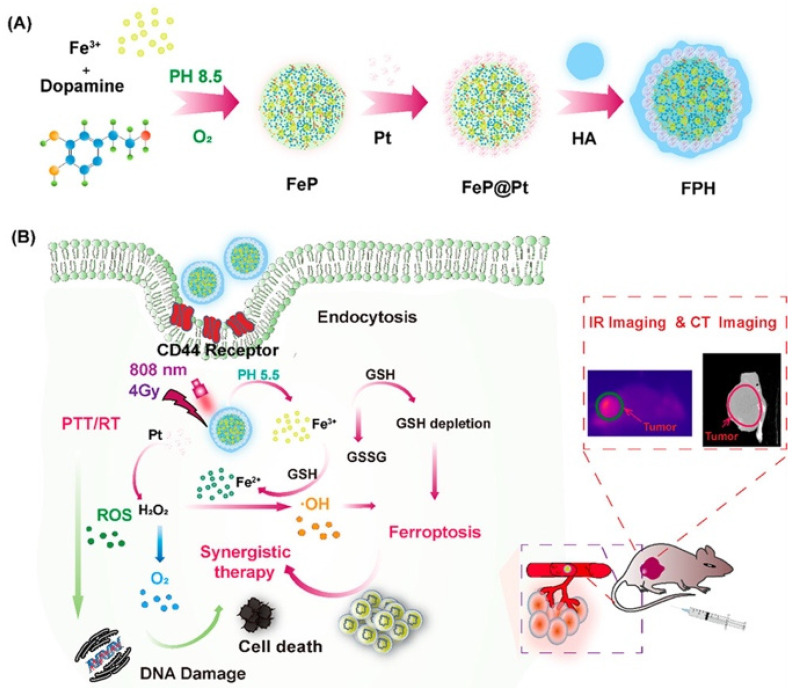
Schematic illustration of the design and therapeutic action of FPH core@shell nanocomposites for multimodal breast cancer treatment. (**A**) Fabrication process of FPH nanocomposites. (**B**) Synergistic interactions between ferroptosis, photothermal therapy (PTT), and radiotherapy (RT) enabled by the FPH platform. Reproduced from [172].

A multifunctional core–shell nanoplatform, FeP@Pt@HA (FPH), was developed for breast cancer therapy, combining dual-modal imaging (computed tomography and infrared thermal) with multimodal therapeutic effects (Figure 5) [172]. Structurally, FPH consists of a Fe(III)-polydopamine core, a Pt nanoshell to enhance CT contrast and generate oxygen for radiosensitization, and a hyaluronic acid coating for CD44-mediated tumor targeting and improved biostability. Upon NIR irradiation, FPH induced photothermal and ferroptosis effects, enabling synergistic tumor ablation. In vivo studies confirmed strong tumor accumulation, low toxicity, and effective imaging-guided therapy, positioning FPH as a promising theranostic platform [172]. Smart Au NPs-stabilized microbubbles (SAuMBs) offer a versatile theranostic platform combining ultrasound imaging, photoacoustic imaging (PAI), and PTT [173]. In acidic tumor environments, pH-responsive gold NPs (SAuNPs) aggregate, enhancing imaging contrast and heat generation under NIR laser irradiation. SAuMBs enable targeted delivery via ultrasound-triggered sonoporation, with efficient SAuNP uptake by tumor cells. In vivo, this strategy led to complete tumor ablation and high survival rates, with no systemic toxicity observed [173].

Ultrasmall superparamagnetic iron oxide NPs (USPIOs) functionalized with polyethylenimine (PEI) have been developed as a dual-function platform for gene delivery and magnetic resonance imaging (MRI) [174]. These USPIO-PEI conjugates form compact polyplexes with plasmid DNA, offering protection against nuclease degradation and efficient cellular transfection with low toxicity. Importantly, the system enables real-time, non-invasive monitoring of gene vector unpackaging via T2 relaxation changes in MRI. This “magnetic relaxation switch” reflects DNA release, making USPIO-PEI a promising tool for image-guided gene therapy [174]. Furthermore, functionalized AuNPs have been developed as effective CT contrast agents [175]. A one-pot synthesis method produced PEG-coated AuNPs with varied morphologies, ensuring colloidal stability and prolonged circulation. CT imaging performance was primarily influenced by surface chemistry rather than shape or size. PEGylated AuNP6 (spherical) and AuNP9 (star-shaped) exhibited extended blood circulation and strong X-ray attenuation. Additionally, glucosamine-functionalized AuNP7 enabled targeted imaging by selectively accumulating in inflamed lung tissue, highlighting their potential for both anatomical and functional CT diagnostics.

#### 4.1.3. Antimicrobial and Antioxidant Applications

NFs and NPs have significantly demonstrated high potential in antimicrobial activity, representing an alternative to traditional antimicrobial compounds, thus complementing the rising concern about antibiotic resistance. Ag NPs are particularly valuable for biomedical applications due to their potent antimicrobial and antioxidant properties [176]. Their antibacterial effects are driven by multiple mechanisms, including the induction of oxidative stress, disruption of DNA replication, and interference with essential microbial proteins, making them effective even against drug-resistant strains [177]. Additionally, their antioxidant activity arises from their ability to scavenge reactive oxygen species (ROS), supporting their potential use in cancer therapy and the treatment of neurodegenerative diseases [177].

Vazquez-Muñoz et al. [178] synthesized Ag NPs via chemical reduction that showed broad-spectrum antibacterial activity with MICs of 10–12 µg/mL. When combined with antibiotics, Ag NPs exhibited synergistic effects with kanamycin (FICI ≤ 0.5) and additive effects with chloramphenicol (FICI 0.5–1) against *E. coli*, *S. typhimurium*, and *S. aureus*, leading to up to 95% growth inhibition. No synergy was observed with β-lactam antibiotics or in *B. subtilis*. Mechanistic studies revealed that Ag NPs disrupt bacterial membrane integrity, enhancing antibiotic uptake. In addition to their strong antimicrobial activity, Ag NPs also demonstrate excellent biocompatibility, which further enhances their potential for biomedical applications. For instance, while Ag NPs showed cytotoxic effects at concentrations ≥2 µg/mL, they were found to be biocompatible at 1 µg/mL [179]. At this safe concentration, Ag NPs alone exhibited no significant antibacterial activity. However, when combined with antibiotics, they displayed potent synergistic effects against both Gram-positive and Gram-negative bacteria, including resistant strains such as *S. aureus*, *S. mutans*, and MRSA. The presence of Ag NPs restored or improved antibiotic sensitivity in several pathogens, suggesting a broad and non-specific mechanism, likely related to increased bacterial membrane permeability [179]. Similar results can be found on [180,181,182].

### 4.2. Biomedical Applications of Green Nanofluids

#### 4.2.1. Targeted Drug Delivery and Cancer Therapy

**Figure 6:**
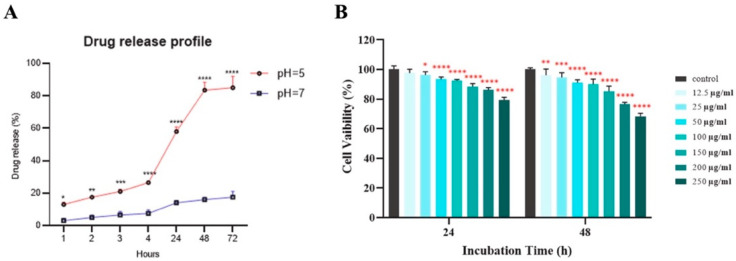
(**A**) Drug release profiles of imatinib loaded in FA–CS–Ag-I at normal and acidic pHs (7.0 and 5.0). After 24 h of release, at pH 5.0, more than 80% of imatinib was release, whereas at pH 7.4, less than 20% of release was achieved. (**B**) Cell viability of MCF-7 cells exposed to various concentrations (12.5–250 μg Fe/mL) of sumac-based superparamagnetic Fe_3_O_4_ NPs at 24 h and 48 h periods of incubation, * *p* < 0.05, ** *p* < 0.01, *** *p* < 0.001, **** *p* < 0.0001 Adapted from [183,195].

Green NFs have been extensively explored as targeted drug delivery systems, particularly in cancer therapy. These NFs may consist entirely of green-synthesized NPs or incorporate green NPs into more complex composites. For example, Ag NPs synthesized from *Spirulina platensis* were integrated into folic acid-functionalized chitosan NPs loaded with imatinib (FA-CS-Ag-I), demonstrating pH-responsive drug release matching the acidic tumor environment and reducing cancer cell viability by ~20% (Figure 6A) [183].

Moreover, ZnO NPs produced via green methods have shown broad anticancer efficacy against osteosarcoma, liver, colon, breast, and lung cancers [184,185,186,187,188,189,190,191,192]. Their small size (with enhanced surface area and reactivity) and bioactive plant-derived phytochemicals enhance cytotoxicity, as exemplified by ZnO NPs (~66 nm) synthesized using *Raphanus sativus* leaf extract, which induced dose-dependent apoptosis in A549 lung cancer cells with an IC_50_ of 40 µg/mL [193]. Hybrid systems such as PEGylated Ag-decorated graphene nanocomposites synthesized using neem leaf extract have also shown promise. These composites improved doxorubicin loading (218%) and exhibited synergistic cytotoxicity against HaCaT (a type of human keratinocyte cell line found in normal skin cells) and HeLa (cancer cell line derived from cervical cancer cells) cell lines. PEGylation enhanced biocompatibility and reduced toxicity toward normal cells, highlighting the balance between efficacy and safety [194].

Biogenic iron oxide NPs, synthesized rapidly using Rhus coriaria extract, demonstrated moderate antiproliferative effects on MCF-7 breast cancer cells [195]. In vitro cytotoxicity revealed reduced toxicity towards MCF-7 breast cancer cells with moderate, dose-dependent anti-proliferative activity, maintaining high cell viability even at elevated concentrations (200 μg/mL and 250 μg/mL for 48 h) (Figure 6B). They also inhibited cancer cell migration in wound healing assays, indicating strong anti-metastatic potential of the MCF-7 cells [195].

Fe_3_O_4_ NPs synthesized using *Garcinia mangostana* peel extract (~13.4 nm) formed NFs with hydrodynamic sizes below 177 nm, showing significant hyperthermia potential and selective cytotoxicity toward HCT116 colon cancer cells (IC_50_~100 µg/mL) over normal CCD112 cells (IC_50_~141 µg/mL) [196]. Similarly, hybrid nanocomposites combining *Moringa oleifera*-derived Fe_3_O_4_ NPs with watermelon rind carbon dots (~8.6–9 nm) demonstrated tunable magnetic and luminescent properties, suitable for drug delivery, magnetic hyperthermia, and imaging [197]. PTT also benefits from green-synthesized NFs. Spinel ferrite NPs (CoFe_2_O_4_ and ZnFe_2_O_4_) produced with rutin extract exhibited superparamagnetism and particle sizes of 29 nm and 25 nm, respectively [198]. The NPs enhanced photothermal effects under laser irradiation in MCF-7 breast cancer cells, highlighting their potential for combined therapy and diagnostics [198]. Additionally, green-synthesized Au NPs (~18 nm) using chitosan as a reducing and capping agent showed synergistic chemo-photothermal activity [199]. While free 6-mercaptopurine (6MP) had minimal effect (~5% inhibition), 6MP-loaded Au NPs achieved up to 63% cancer cell inhibition under laser exposure (532 nm), attributed to improved drug uptake and photothermal conversion disrupting DNA repair [199]. 

#### 4.2.2. Theranostics and Imaging

Beyond therapy, green NFs contribute to the field of theranostics, which is a strategy where a single agent can be used for both therapeutic and diagnostic solutions. This dual functionality is advantageous since it can allow physicians to monitor the delivery and effectiveness of the therapy while simultaneously providing therapeutic treatment, reducing side effects, and improving target specificity [200]. 

Composite IO–Au NPs were synthesized using an aqueous extract of *Pimenta dioica* leaves, yielding NPs with an average diameter of approximately 19 ± 3 nm [201]. Relativity studies performed on a clinical 1.5T magnetic resonance imaging (MRI) scanner demonstrated that the IO-Au NPs have high relaxivity values (r1 = 10.67 mM/s and r2 = 140.87 mM/s) with an r2/r1 ratio of 13.20. The findings confirm that the green NPs can serve as effective T2 contrast agents, thereby improving the quality of MRI images. Ex vivo imaging experiments with chicken tissue and poultry heart were found to demonstrate strong proof of contrast improvement, with the treated tissues exhibiting much darker T2-weighted images compared to control samples. Furthermore, IO-Au NPs exhibited strong photothermal properties, with temperature increases dependent on both concentration and laser power. At a high concentration (3.0 mg/mL), the NPs raised temperatures by up to 13.1 °C under 42 W/cm^2^ NIR irradiation, which is significantly higher than water controls (2.9 °C) [201]. The results confirm the potential of these green-synthesized NPs for controlled, concentration- and power-tunable photothermal cancer therapy and imaging. Furthermore, composite NPs of CoFe_2_O_4_/ZnS synthesized using *Moringa oleifera* extract showed SAR (specific absorption rate) enhancement from 87.8 to 132.9 mW/g as ZnS content increased, making them potent candidates for magnetic hyperthermia [200]. The inclusion of ZnS NPs imparts optical properties that benefit fluorescence imaging, helping in both diagnosis and real-time tracking of treatment, suggesting their role as multifunctional agents in image-guided therapy.

#### 4.2.3. Antimicrobial and Antioxidant Applications

While the antioxidant activity of Ag NPs has already been demonstrated before, green-synthesized Ag NPs (particularly using plant extracts rich in bioactive compounds such as polyphenols and flavonoids) appear to exhibit enhanced antioxidant potential. These compounds enhance the NPs’ ability to neutralize reactive oxygen species, contributing to applications in cancer therapy and neurodegenerative disease treatment. Notably, green-synthesized Ag NPs often show improved antioxidant and cytotoxic effects while maintaining biocompatibility with normal cells. This dual functionality supports their growing use in pharmaceuticals, wound healing, and therapeutic formulations.

Green-synthesized Ag NPs have demonstrated broad-spectrum antimicrobial and anticancer properties, with performance largely influenced by the natural source. Ag NPs derived from *Neurada procumbens* (~93 nm) exhibited antibacterial activity (15–17 mm inhibition zones against *S. aureus* and *B. cereus*) and induced oxidative stress, cell cycle arrest, and apoptosis in MCF-7 and HeLa cancer cells [202]. Smaller Ag NPs (10–40 nm) synthesized from *Argyreia nervosa* leaves also showed strong antibacterial effects against *E. coli* [203]. Comparative studies of Ag NPs from lemon, black seeds, and flax revealed variable antimicrobial activity against drug-resistant pathogens [204]. Lemon-derived Ag NPs (L-AgNPs) exhibited the highest activity, especially against Gram-positive bacteria and *Candida albicans*, whereas black seed (B-AgNP) and flax-based (F-AgNP) counterparts showed limited efficacy, targeting only *Enterobacter cloacae* [204]. However, some pathogens, including *E. coli* and *S. aureus*, exhibited resistance to all tested NPs, underscoring the complexity of green Ag NP bioactivity and the need for further optimization [204]. Furthermore, Ag NPs from *Acacia sinuata* exhibited dual mosquitocidal and anticancer effects, with significant cytotoxicity against Caco-2 cells (IC_50_ = 1.03 µg/mL), suggesting potential in both vector control and oncology [205].

*Podocarpus macrophyllus* leaf extract was used to synthesize Ag NPs with multifunctional therapeutic potential, including strong antioxidant (90% radical scavenging), anti-inflammatory (99.15% protein denaturation inhibition), anti-diabetic (90.56% α-amylase inhibition), and anti-hemolytic (89.9%) properties [206]. These NPs also exhibited potent antibacterial activity against *Staphylococcus* spp. (20 mm inhibition zone) [206]. In silico studies suggested potential interaction with the NOTCH2 gene, frequently upregulated in gliomas, supporting possible applications in brain cancer therapy [206]. Similarly, green-synthesized ZnO NPs have shown outstanding antimicrobial performance. *Magnolia officinalis*-derived ZnO NPs achieved nearly complete inhibition (99.99%) of *E. coli* and *S. aureus*, and retained this activity after six months of storage, highlighting their stability and potential for long-term use [207]. Additionally, ZnO NPs synthesized with *Loranthus cordifolius* extract and loaded with anethole demonstrated enhanced antimicrobial effects against both Gram-negative (*Pseudomonas aeruginosa*, *E. coli*) and Gram-positive (*Bacillus subtilis*, *S. aureus*) bacteria compared to commercial ZnO NPs, suggesting a synergistic benefit from plant-derived compounds [208].

Finally, green-synthesized Fe_2_O_3_ NPs have also shown remarkable antimicrobial efficacy. Biogenic Fe_2_O_3_ NPs produced using *Cissus rotundifolia* exhibited strong antibacterial action against *P. aeruginosa* and *K. pneumoniae*, with time–kill assays confirming bactericidal activity [209]. These NPs also displayed potent antioxidant (89.53% DPPH inhibition) and anti-inflammatory effects (81% inhibition in BSA assay). These multifunctional capabilities support their use in wound healing, infection control, and broader therapeutic applications [209].

### 4.3. Comparative Insights into Green and Conventional Derived Nanofluids for Biomedical Applications

Although NFs show significant potential in biomedical use, direct comparisons involving chemically synthesized and green-synthesized NFs are scarce. Since the performance of NFs is not only route-dependent but also on essential NP properties (size, charge, shape, and concentration), it is difficult to draw a conclusion based on independently conducted research.

To offer a more unbiased evaluation, this section presents data from studies of direct comparisons between green and chemically synthesized NFs under similar or standardized conditions, as illustrated in Table 1. In most cases, green-synthesized NFs tend to be more biocompatible, less cytotoxic, and more bioactive, and reduce environmental footprints by avoiding toxic chemicals and energy-consuming processes [210,211,212,213]. However, such advantages are not absolute. In some cases, NFs prepared chemically have greater colloidal stability or smaller size distributions with enhanced pharmacokinetics control and biodistribution [214]. Moreover, variability in the composition of plant/algae extracts and the lack of standardized synthesis protocols in green approaches can affect reproducibility and scale-up feasibility.

## 5. Heat Transfer Applications of Nanofluids

NFs stand out for their exceptionally large specific surface area and superior thermal conductivity, which together amplify heat transfer between fluids and NPs and boost the performance of high-temperature systems—whether in heat pipes, heat sinks, exchangers, or microfluidic devices [215]. Their nanoscale dimensions not only enable more compact designs and reduce the risk of clogging common in conventional suspensions but also offer the flexibility to tune properties like thermal conductivity and wettability simply by adjusting particle concentration. The following sections examine the experimental results and thermal advantages observed in both conventionally synthesized and green-synthesized NFs. In addition, their increasingly diverse applications in thermal engineering are highlighted, covering solar collectors, electronics cooling, engine thermal management, nuclear reactors, transformers, and other emerging areas.

### 5.1. Heat Transfer Applications of Conventional Nanofluids

#### 5.1.1. Solar Panels and Collectors

Photovoltaic (PV) panels convert solar energy into electricity via photovoltaic cells, which are sensitive to temperature. Elevated surface temperatures, often due to excessive solar radiation, degrade PV efficiency, typically by 0.4–0.5% per °C above the optimal 25 °C [216]. This challenge is particularly significant in warmer climates, prompting research into advanced cooling strategies, notably using NFs.

**Figure 7:**
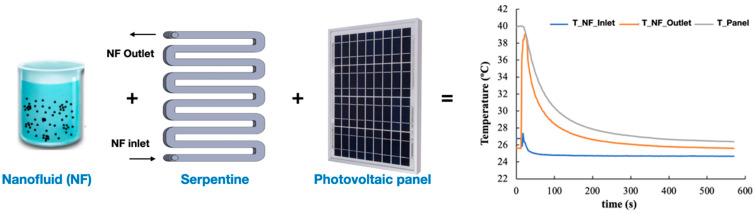
Scheme illustrating the use of NFs in serpentines for cooling photovoltaic solar panels.

Recent studies have primarily explored serpentine heat exchangers integrated with PV/thermal systems using NFs [217,218] (Figure 7). These configurations influence the superior thermal conductivity of NPs to enhance heat dissipation and lower cell temperatures. Ebaid et al. [216] evaluated Al_2_O_3_ (431 nm) and TiO_2_ (50 nm) NFs at concentrations up to 0.1%, comparing their cooling performance against water and ambient air for 50 W PV modules. The results showed improved electrical efficiencies of 13.8% (TiO_2_ NF) and 14% (Al_2_O_3_ NF) at 3000 mL/min, outperforming both water and air cooling (13.5%). Hussien et al. [219] investigated Al_2_O_3_ (30 nm)–water NFs in a hybrid PV/thermal system under forced convection. A 3% concentration yielded optimal results, lowering panel temperature to 42.2 °C and increasing efficiency to 12.1%. Higher concentrations, however, raised temperatures (52.2 °C) and reduced efficiency (11.3%), highlighting the importance of optimizing NP concentration to balance heat transfer gains against pressure losses and pumping requirements.

Singh Rajput et al. [220] conducted an experimental investigation on a flat-plate solar collector with Al_2_O_3_ (10–15 nm) NPs dispersed in distilled water using a surfactant SDS (0.8 wt.%) for enhanced stability. They experimented on 0.1–0.3% volume concentrations on a 1.3 m × 2.3 m collector at 27° inclination and recorded an appreciable efficiency gain of 21.32% at 0.3% concentration with the thermal conductivity of 0.622 W/m·K.

Michael and Iniyan [221] investigated CuO/water Sodium Dodecyl Benzene Sulfonate (SDBS)-stabilized NFs at a low concentration of 0.05%. The NPs, synthesized by aqueous precipitation of Cu acetate, were evaluated in a 2.08 m × 1.05 m collector and demonstrated an enhanced thermal efficiency by 6.3% under a 0.1 kg/s flow rate. Moreover, Colangelo et al. [222] reported NP sedimentation of Al_2_O_3_ (45 nm), ZnO (60 nm), and Fe_2_O_3_ (30 nm) NFs at 1–3 vol.% in flat-plate collectors with an inclination angle of 30°. The results emphasized the effect of fluid velocity on stability. Thermal conductivity increased by 6.7% (to 0.722 W/m·K), and convective heat transfer coefficient increased by 25% for 3 vol.% Al_2_O_3_, showing the compromise between enhancement of performance and potential settling at high concentration.

Thermal absorption capacity of NFs is among the most significant features of NF behavior in solar thermal systems. An experimental study comparing carbon nanotube, Cu, and Al-based NFs (in water) revealed that increased concentrations of NPs)enhanced absorbance as well as electrical conductivity, both of which directly improved thermal absorption [223]. This is significant, given the strong correlation between solar energy efficiency and the optical properties of the working fluid [224]. Sidik et al. [225] emphasized that the combined improvements in light absorption and thermal conductivity make NFs especially well-suited for solar collectors.

Direct Absorption Solar Collectors (DASCs), initially created in the 1970s by Minardi and Chuang, have recently gained attention because of their ability to enhance thermal performance via the working fluid, acting as absorber and heat transfer fluid, minimizing heat loss [226]. High optical absorption, high thermal conductivity, and photothermal conversion efficiency are required in fluids to provide maximum DASC performance. Chen et al. [227] tested reduced graphene oxide (RGO)/water NFs, which were prepared through UV irradiation of graphene oxide suspensions, in DASCs. The NFs showed superior stability, optical absorption, and thermal conductivity. Notably, RGO-based NFs demonstrated the best photothermal conversion efficiency (96.93% at 30 °C, 52% at 75 °C) compared to graphene oxide and graphene NFs, making them an ideal choice for low-temperature DASC application.

Ahmad et al. [228] highlighted the limited research on the optical properties of NFs. Their work emphasized critical observations, namely (1) solar absorption rises with increasing NP size and volume fraction; (2) the absorption is extremely sensitive to optical path length; (3) transmittance reduces for increasing particles, concentrations, and travel distances; (4) light scattering depends directly on NP size and concentration; and (5) aggregation of bigger particles increases scattering and the extinction coefficient. Moreover, Ahmad et al. [228] pointed out that, in addition to enhanced thermal conductivity, several often-overlooked factors influence NF performance in solar systems. They include homogeneous transmission of received energy to prevent thermal hotspots and reduce heat losses [229]; pressure losses due to increased NF viscosity at elevated concentrations; and the requirement for inexpensive NP synthesis procedures. They also requested a significant research gap in experimental investigations on the use of NFs in non-traditional solar devices such as solar thermoelectric cells, solar ponds, parabolic trough collectors, and photovoltaic–thermal (PV/T) systems. Experimental verification at a detailed level is crucial to understand and optimize NF performance comprehensively in a range of solar energy applications.

#### 5.1.2. Electronics Cooling

**Figure 8:**
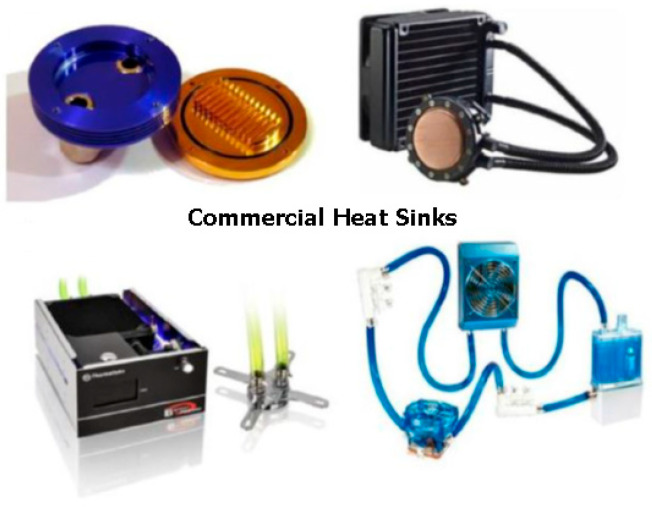
Examples of commercial heat sinks for different kinds of electronic systems, adapted from [231].

Owing to technical advancements, electronic devices are increasingly compact and lightweight, while their processing speed has also improved. Furthermore, it can be determined that these devices produce higher heat fluxes, and conventional air-based cooling technologies are inadequate for effective heat dissipation [230,231]. Figure 8 illustrates various examples of commercial heat sinks utilized in liquid-based processing units (CPU) coolers.

The recent developments of electronic heat management, and more precisely CPUs, have put NFs in a position as better alternatives to conventional base fluids (BFs) due to their enhanced performance in heat transfer. Most research on commercial cooling systems always reiterates the performance betterment of NFs. Korpyś et al. [232] evaluated a CuO–water NFs (0.86–2.25 vol.%) formulated in a double-step process from commercial CuO NPs (30–50 nm) with diammoniumhydrocitrate stabilizer for a ZMWB3 Au heat sink. At 2.25 vol.%, the NF reduced CPU temperature by 0.5 °C compared to water alone. Turgut et al. [233] reported a 6.7% contact surface temperature reduction (from 40.2 °C to 37.5 °C) by Al_2_O_3_ NPs (10 nm) with a CoolerMaster Seidon 120M system. Roberts et al. [234] achieved an 18% rise in convective HT—to 3191 W/m^2^K—using a 1.5 vol.% Al_2_O_3_–water NF in the ThermalTake Bigwater 760 system. Rafati et al. [235] also evaluated 3D Galaxy II on a AMD Phenom II X4 965 CPU, with SiO_2_, TiO_2_, and Al_2_O_3_ NFs compared. Al_2_O_3_ outperformed the others under all conditions, lowering CPU temperature from 49.4 °C to 43.9 °C due to its superior HT coefficients at comparable flow rates and concentrations.

Innovative heat pipe CPU cooling designs have also obtained the maximum advantages of NF addition. Heat pipes commonly contain an evaporator and condenser connected by microchannels filled with coolant. Yousefi et al. [236] theoretically studied the effect of inclination angle and NF content on heat pipe performance using MKN-Al_2_O_3_-G015 NPs as a surfactant with SDBS. Reducing the inclination angle from 60° to 30° increased CPU temperatures, but the addition of 0.5 wt.% Al_2_O_3_ NPs decreased thermal resistance by 15% at 10 W and 22% at 25 W, proving the feasibility of thermal improvement using NFs under such a configuration.

In optical and microfluidic devices, where small volumes of fluid must be precisely controlled, NFs have been more efficient under electric fields than the base fluids. Dash et al. [237] demonstrated increased electrowetting response with bismuth telluride NPs in water. When a droplet was actuated on a Teflon-coated silicon wafer, NFs lowered contact angle more than base fluids, being useful in applications like lab-on-chip systems and display technologies with controlled, high-speed fluid motion. 

NFs thus present a highly promising solution to high-end heat management in a range of applications, from consumer products to microfluidics, phase change systems, and defense technology. Their higher thermal conductivity and specific heat capacity make them eligible for high heat flux applications. However, for issues like increased viscosity and associated pressure drops, more optimization is required. While encouraging experimental results [238,239], large-scale consumer applications are not yet underway, although ongoing research and further technical advancements bring the field closer to practical realization in commercial as well as in critical military uses.

#### 5.1.3. Engine Cooling

Effective thermal management is vital for engine performance and has effects on both fuel economy and emissions of pollutants. Effective heat control systems are essential in today’s vehicle architecture [240]. Radiators, being significant heat exchangers, dissipate waste engine heat. However, increased engine power demands and Exhaust Gas Recirculation (EGR) system provisions have led to increased radiator size with increased frontal area and accompanying aerodynamic drag and fuel consumption [241]. Also, indicating that thermal management remains a problem due to the limited thermophysical properties of conventional coolants and inefficiencies of heat exchanger surface design.

Microfabrication technology and NFs hold potential solutions. With enhanced thermal conductance of engine coolants and lubricants, NFs enable more efficient, lighter, and compact radiators, which contribute to better fuel economy and reduced vehicle weight [242]. Conventional coolants in the form of water/ethylene glycol mixtures are less effective than water; incorporating NPs can significantly enhance heat transfer. Saidur et al. [243] illustrated that CuO nanofibers effectively lowered engine temperatures at different speeds. Nevertheless, problems like NP sedimentation, agglomeration, and surface erosion need to be addressed with caution. Leong et al. [244] reported that greater Cu NP concentrations elevated pressure drops, i.e., a 2 vol.% Cu NF produced a pumping power increase of 12.13% compared to the base fluid. NF properties are also temperature-sensitive: higher inlet temperatures reduce viscosity and internal fluid shear, which lowers frictional losses [245]. Still, NF performance is not always consistent. Kulkarni et al. [246], operating with diesel generators, noted that the cogeneration efficiency decreased from 79.1% (50:50 EG/water mixture) to 76.1% with 6% Al_2_O_3_ NPs owing to the reduction in specific heat capacity (~3000 J/kg·K to ~2500 J/kg·K).

While the results are promising, some obstacles remain. One of the biggest concerns is the absence of extensive research on the tribological behavior of NFs and the long-term erosion of radiator material due to suspended NPs. It is essential to overcome these shortcomings by predictive modeling and prolonged durability testing to move forward with the sustainable deployment of NFs in automotive thermal systems.

#### 5.1.4. Nuclear Reactors

Nuclear reactors represent a low-carbon option for mass power production that has the potential to decrease the world energy supply by as much as 17% by the year 2050 [247]. However, their use is also associated with high thermal energies and stringent safety conditions. For such an application, NFs are a viable method of enhancing HT and safety margins. Rahnama and Ansarifar [248] simulated incorporation of an Al–water NF into a NuScale reactor, a Small Modular Reactor (SMR) with passive safety and natural circulation cooling. Initial concerns over NF incompatibility with natural convection-based systems were alleviated as their simulation showed that alumina NPs (26.174 nm, 0.828 vol.%) improved buoyancy-driven flow and thermal performance. Buongiorno and Hu [249] presented a comprehensive study of the improvement of critical heat flux (CHF) contributed by NFs to nuclear systems. In comparison with alumina, zirconia, and silica NPs (≤0.1 vol.%), they attained a CHF of as high as ~2.4 × 10^5^ W/m^2^ using alumina in pool boiling. Their results expanded the CHF dataset relevant to reactor conditions and suggested that the application of NFs can reduce system failure probabilities through better coolant distribution. Most notably, high-power-density light water reactor vessel-rupture safety margins increased by ~40% between NF applications. The study also evaluated alumina nanofiber colloidal stability after gamma radiation and chemical exposure (boric acid, lithium hydroxide) with high durability. Despite such advances, fundamental problems persist. Thermal–hydraulic performance of NFs under operating reactor conditions is still not adequately researched. Furthermore, detailed analyses of chemical compatibility of NFs with structural materials in the reactor have to be carried out to ensure long-term viability [250].

#### 5.1.5. Space Technology

Due to weight and space limitations, along with energy on space and air vehicles, compact yet highly heat transfer efficient systems have gained much attention [251]. NFs, being capable of sustaining extremely high heat fluxes, hold much promise in miniaturizing thermal management systems employed in aerospace [252]. Microgravity is a serious problem, though, in their use. Several numerical studies have used NF behavior in microgravity to resolve this problem. Chen et al. [253] investigated thermocapillary convection in 2D cavities with graphene-based silicone NFs with an optimal volume concentration of 3%. Above this, the efficiency of convection and the velocity of the free surface decreased. Yanoaka and Inafune [254] confirmed that for low gravity fluctuations, natural convection was increased by 1% vol. NF but decreased by larger gravity fluctuations and higher concentration of NP to 5%. Kamal et al. [255] reported that needle-like metallic NPs showed as much as a 14% enhancement in heat transfer compared to other shapes, and the NP material also contributes to flow behavior. Das et al. [256] proposed the integration of NFs into active thermal control systems (ATCSs), which are crucial in spacecraft electronics and habitat temperature control. Ungar and Erickson [257], through parametric modeling, demonstrated that NFs could significantly reduce the size, weight, and pumping power of ATCS components. Aside from fluid cooling, Kuo et al. [258] employed hydroxyl-terminated polybutadiene (HTPB) solid fuel with added Al NPs and demonstrated that the linear regression rate improved by 123%, significantly improving the specific energy output of rocket propellants. This highlights the multilateral advantage of NPs in thermal management and propulsion in aerospace applications.

#### 5.1.6. Transformers

Proper cooling is vital for transformers, particularly as rising global electricity needs dictate that they be upgraded or replaced, often at great expense. Replacement of conventional transformer oils with NFs offers a worthwhile alternative with improved thermal characteristics. Choi et al. [259] proved that dispersion of AlN (50 nm) and Al_2_O_3_ (13 nm) NPs into transformer oil with oleic acid as a dispersant improved thermal conductivity by 8% and the total heat transfer coefficient by 20% at a very low content of 0.5 vol.% AlN, using a transient hot-wire technique. It can improve transformer life and cooling performance. However, dielectric performance is of concern. Conductive NPs like Fe_2_NiO_4_, Fe_2_O_3_, and Cu can reduce dielectric strength [260,261,262], whereas ZnO and CuO have been reported to have beneficial effects [263].

Thermal aging resistance is also required. Segal et al. [264] stated that dielectric ferrofluid mixed with mineral oil remained stable in performance for 50 weeks at 185 °C. Mergos et al. [265] investigated various NPs (Al_2_O_3_, TiO_2_, Fe_2_O_3_, CuO, Cu_2_O) dispersed in paraffin oil up to 5% *w*/*v* and stated that grain size, chemical composition, and water adsorption were significant parameters influencing dielectric properties. The reported dielectric improvements are due to internal polarization, particularly in ZnO-based NFs NFs [266]. Top-oil and hot-spot temperatures have been lowered by ~5 °C with the incorporation of Fe_3_O_4_ NPs [267], allowing smaller, more efficient transformers [268], and improvements in insulation, moisture resistance, and thermal aging [269,270]. Yet, challenges such as altered electrical conductivity, permittivity, and loss factor (including affecting the stress distribution) still hinder the large-scale adoption of NFs in transformers [265,271].

#### 5.1.7. Heat Pipes

Micro heat pipes are widely used in miniature systems, and NFs proved to be effective as working fluids in these applications [272]. Among them, pulsating heat pipes (PHPs)—pressure-difference-driven, wickless systems—are very appealing for NF integration. Li et al. [273] compared a stable water-based TiO_2_ NF (up to 2 wt.%) for electric vehicle battery cooling during routine and hot charging. The NF improved the temperature homogeneity by 82% (temperature variation < 1 °C throughout the module) and reduced temperature rise by up to 80% compared to natural convection, thus enhancing charging efficiency. Zhou et al. [274] compared PHP startup performance with pure water and graphene oxide (GO) NFs. The highest performance was achieved using 0.05 wt.% GO at a 50% filling ratio, which caused a 34 °C decrease in startup temperature and a 58% decrease in startup time. Based on their research, Afsari et al. [275] used GO (10–50 nm) along with SDS and Triton X-100 surfactants in thermosyphon heat pipes and lowered the evaporator temperature by 12.3 °C through the use of 0.3 wt.% GO in water. In addition, higher working temperatures still improve oscillating heat pipe effectiveness, making NFs a viable innovation for miniaturized cooling systems [276].

Overall, NFs provide revolutionary advances in automotive thermal management by boosting heat transfer, reducing radiator size, and improving fuel economy. Widespread application, however, depends on the ability to overcome material compatibility issues and optimize nanofluid formulations to enhance performance while ensuring long-term system integrity.

### 5.2. Heat Transfer Applications of Green Nanofluids

#### 5.2.1. Solar Panels and Collectors

**Figure 9:**
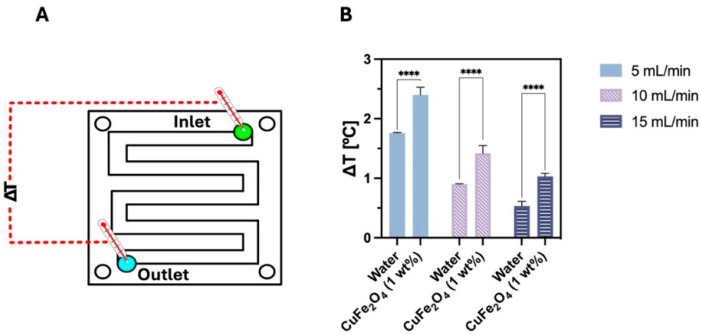
(**A**) Diagram showing how temperature differences between the inlet and outlet were measured. (**B**) Measured temperature differences across the serpentine channel for water and 1 wt.% CuFe_2_O_4_ nanofluid at flow rates of 5, 10, and 15 mL/min. Values are shown as mean ± standard deviation from three separate experiments. Error bars reflect standard deviation. Statistical analysis was performed using ANOVA with Šídák’s post hoc test. **** *p* < 0.0001. Reproduced from [278].

Although NFs have been extensively investigated for thermal applications, green NFs remain underexplored concerning their thermal properties. Nonetheless, recent studies have demonstrated their considerable potential. Nobrega et al. [277] synthesized Fe_3_O_4_ NFs using the microalga *Chlorella vulgaris* and formulated a water-based NF. At a concentration of just 0.1%, this green NF exhibited a thermal performance enhancement of 6.4% compared to the base fluid under identical testing conditions. In a similar experimental setup, Cardoso et al. [278] developed a Cu ferrite-based NF, also synthesized via *C. vulgaris*. Figure 9 shows that the CuFe_2_O_4_ (1 wt.%) NF achieves a higher heat transfer rate than water, especially at increased flow rates, with statistically significant enhancement observed only at 15 mL/min. On average, the NF stores 38% to 95% more heat than water, highlighting its superior cooling potential for thermal applications.

In another study, α-MnO_2_ NPs were synthesized using Ficus retusa leaf extract and incorporated into capric acid to form a phase change material (PCM) [279]. The resulting composite demonstrated excellent energy storage capacity, retaining performance even after 500 thermal cycles. This highlights the material’s reliability and thermal conductivity enhancement due to accelerated charging and discharging rates.

The application of green NFs is the solution for optimizing the thermal regulation of photovoltaic (PV) panels and solar collectors. Green-synthesized TiO_2_ and Al_2_O_3_ improved the convective heat transfer coefficient of working base fluids. This increases the surface temperatures of the PV system and improves its overall efficiency. Hamdan and Kardasi [280] studied water-based TiO_2_ NFs for PV panel passive cooling, clearly demonstrating that significant temperature reduction was accompanied by an improvement in energy efficiency. Another study by Mustafa et al. [281] demonstrated the effectiveness of NFs with phase change materials (PCMs) in cooling applications. The study concluded that adding NFs to the system greatly enhances thermal conductivity without compromising system safety.

Ranjbarzadeh et al. [282] conducted an experimental investigation to assess the influence of SiO_2_ NPs synthesized using rice bran as a plant-based precursor on the thermal conductivity of water. The NFs were formulated using a two-step preparation method and demonstrated excellent long-term colloidal stability, remaining homogeneous and free of sedimentation for over six months. Thermal conductivity measurements were carried out across a temperature range of 25 °C to 55 °C and NP volumetric concentrations of up to 3%. The results indicated a maximum enhancement of 38.2% in thermal conductivity compared to pure water. Furthermore, the improvement in thermal conductivity was found to be positively correlated with both increasing NP concentration and temperature. In turn, gallic acid-functionalized graphene nanoplatelets (GGNPs) NFs at 0.025%, 0.05% and 0.1% by mass, dispersed in distilled water, were applied in a flat-plate solar collector system [283]. The results showed an increase of up to 17.76% in thermal conductivity and an increase of up to 21.48% in collector efficiency when using 0.1% of GGNPs [283]. In addition, the use of green fluid allows to reduce the required collector area by 26.41% and shorten the financial payback period by 5.615% [284].

#### 5.2.2. Electronics and Engine Cooling

As electronic components become more compact and powerful, their thermal loads increase significantly. Green NFs are the advanced cooling media for these systems. They offer high thermal conductivity and specific heat capacity while being environmentally benign. Saidur et al. [243] reviewed NF applications and concluded that their use in electronics significantly improves heat transfer performance compared to conventional coolants. Green NPs are an ideal solution for cooling sensitive electronics. Their colloidal stability and low toxicity make them compatible with such electronics and ensure a reliable and sustainable cooling solution. In engine cooling applications, green NFs synthesized from natural mineral sources such as zircon sand and palm oil waste exhibit excellent stability, low sedimentation, and improved thermal properties. These characteristics support their integration into radiator systems, reducing operational temperatures and enhancing insulation performance [285]. Jebali et al. [286] investigated the thermal conductivity of green NFs formulated with ZnO NPs synthesized using lemon juice as a reducing agent. The NPs were dispersed in a mixture of propylene glycol and water (40:60 *v*/*v*), without the use of surfactants. The experimental results showed a linear increase in thermal conductivity with an increase in temperature (20 °C to 70 °C) and NP concentration of 0.5%, 1.0%, and 1.5% by volume. Thermal conductivity increased by 2.85%, 3.63%, and 5.71%, respectively, for the three concentrations, at 20 °C. The improvement in conductivity was attributed to the breakdown of agglomerates, variations in particle shape, and interfacial effects.

#### 5.2.3. Transformers and Heat Pipes

Green NFs are the obvious sustainable replacement for conventional mineral oil-based fluids in the context of power transformer cooling and insulation. Hao et al. [287] reported that Ag NPs synthesized via *Paramignya trimera* extract have improved thermal conductivity and long-term colloidal stability. These are crucial for maintaining transformer efficiency and reducing operational risk.

Sarafraz et al. [288] used an aqueous extract of fresh tea leaves to synthesize Ag NPs. Ag nitrate was added to the extract, which had a total organic carbon (TOC) content of 17.97 g/L. The solution was stirred for two hours at a temperature between 30 and 50 °C, resulting in spherical Ag NPs with diameters ranging from 40 to 50 nm. XRD results confirmed that the resulting NPs contained virtually no Ag oxide, indicating they were composed of pure metal. Tests conducted using a heat pipe showed that the optimal concentration of Ag NPs in the working fluid (water) was 0.4 wt.%, leading to a reduction in the average temperature of the heat pipe, particularly in the evaporator section, which is the most critical, an improvement in thermal efficiency reaching 95.5 kW/mK, and a decrease in response time.

#### 5.2.4. Other Applications

Green tea (*Camellia sinensis*), extract-based bio-NF was tested using Ag NPs as NPs, suspended in a 50:50 ethylene glycol–water mixture without the use of chemical stabilizers or surfactants to yield an eco-friendly formulation [289]. Thermal conductivity increased by up to 37% at 1 vol.% NP loading, with dispersion stability maintained for 12 days and diameters of particles varying between 40 and 60 nm. Heat transfer coefficient improvements of 22%, 36%, and 67% were observed at 0.1%, 0.5%, and 1% NP loadings, respectively, though pressure drops also increased, indicating moderate hydrodynamic penalties [289]. Sadri et al. [290,291] utilized clove extract as an environmentally friendly alternative to toxic acids to covalently functionalize MWCNTs and GNPs. Functionalized aqueous MWCNTs achieved a 20% thermal conductivity improvement at merely 0.08 vol.%, and with great stability. Moreover, NFs of GNP were examined in turbulent flow at 0.1 wt.% with a 22.92% thermal conductivity improvement and a 37.54% enhancement in heat transfer coefficient with low viscosity and stability for 63 days [291]. Similarly, aqueous *Callistemon viminalis* flower extract was used to prepare CuO nanoplatelets, which were dispersed in deionized water (0.1–9 wt.%) and stabilized with 15% polyvinylpyrrolidone (PVP) [292]. The thermal conductivity improved incrementally by up to 34%. This solvent-free, non-toxic synthesis indicates the future and sustainability of green NFs in thermal applications. 

In summary, green NFs exhibit good thermal performance, high colloidal stability (typically up to 180 days), and clean synthesis from environmentally benign additives. Thermal conductivity enhancements range from 4.8% to 38.2%, depending on NP type, synthesis route, and concentration. Higher NP concentration boosts conductivity due to increased particle–fluid contact and formation of efficient thermal paths. However, they add to viscosity and pressure drop, necessitating performance vs. feasibility trade-offs. Industrial uptake demands technological advances in synthesis, modeling, and validations against actual systems to facilitate scalable and sustainable deployment.

#### 5.2.5. Comparative Insights into Green and Conventional Derived Nanofluids for Heat Transfer Applications

The incorporation of NPs into traditional base fluids enables the development of more efficient, compact, and advanced thermal systems. Currently, development has been directed towards designing thermal materials with environmentally sustainable approaches that offer high thermal performance, while meeting the growing demands for energy efficiency. In this context, green NFs emerge as an alternative, offering not only less environmental impact during manufacturing and disposal, but also significant improvements in thermal conductivity [21,293]. Research shows that green NFs can match or even surpass the thermal performance of conventional NFs, with the added benefit of being environmentally safe, more stable, and less corrosive [282,284,289]. These advancements have driven applications in heat exchange systems, solar collectors, and cooling electronic devices. 

One of the main benefits observed in green NFs is the increased colloidal stability, i.e., the ability of the NPs to remain uniformly dispersed in the fluid for longer periods. This stability reduces the tendency of NPs to agglomerate or sediment, which ensures that thermal performance is maintained over time [21]. Table 2 reinforces this observation by comparing different types of NFs, including those produced through conventional methods and green synthesis routes. The data indicate that both types can provide significant thermal enhancements, with notable increases in thermal conductivity often exceeding 30% depending on the NPs, concentration, and preparation method. Furthermore, several studies report satisfactory levels of colloidal stability.

Despite variations in NP types, synthesis methods, and concentrations used, the data presented in Table 2 clearly reveal a common trend across the studies: NFs consistently show improvements in thermal conductivity compared to traditional base fluids. In many cases, these enhancements exceed 30%, highlighting the strong potential of these materials for heat transfer applications. Moreover, it is observed that both conventionally synthesized NFs and those produced through green synthesis routes can achieve good colloidal stability, particularly green NFs, which often demonstrate prolonged stability, with reports of uniform dispersion lasting over 60 or even up to 180 days, depending on the formulation.

## 6. Machining Applications of Nanofluids

Machining operations, including turning, milling, drilling, and grinding, have particular thermal and mechanical needs, such that they require effective lubrication and cooling for tool life and surface integrity [299]. Turning has a rotating workpiece with a single-point cutting tool, where localized heat is generated. Milling has a rotating multi-edge tool on a fixed workpiece, where cyclic thermal loading is generated. Drilling, because of constrained cutting, requires effective chip removal and cooling. Grinding is an abrasive high-precision operation with high specific energy requirements [300].

Cutting fluids lubricate, cool, and evacuate chips, improve surface finish, tool life, dimensional tolerance, and overall efficiency [301]. Conventional fluids raise health, environmental, and cost concerns, constituting roughly 16–20% of total manufacturing expenditures [302,303]. Alternative methods like dry machining and minimum quantity lubrication (MQL) offer cleaner alternatives. MQL uses concentrated, minimum lubrication, reducing fluid consumption but not sacrificing or diminishing performance, in line with clean manufacturing principles [18,304]. NFs enhance the process of heat transfer and reduce friction between the cutting interface. NPs form a lubricant film that lowers tool wear, cutting forces, and burr formation and hence improves surface integrity [305]. Cutting fluids are broadly classified as plain oils, water-soluble fluids, and gaseous coolants [306], of which the water-soluble fluids can be used for NF preparation because they are compatible with colloidal suspensions [307]. NPs (metal, non-metal, or carbon) improve base fluid thermal conductivity, lubricity, and rheological properties without compromising stability or pressure drop [308]. NPs size diminution and higher concentration improve thermal conductivity [146,309,310], which can provide higher heat extraction and enhance tool life. Graphite and MoS_2_ solid lubricants significantly minimize friction, surface roughness, and cutting forces [311,312].

**Figure 10:**
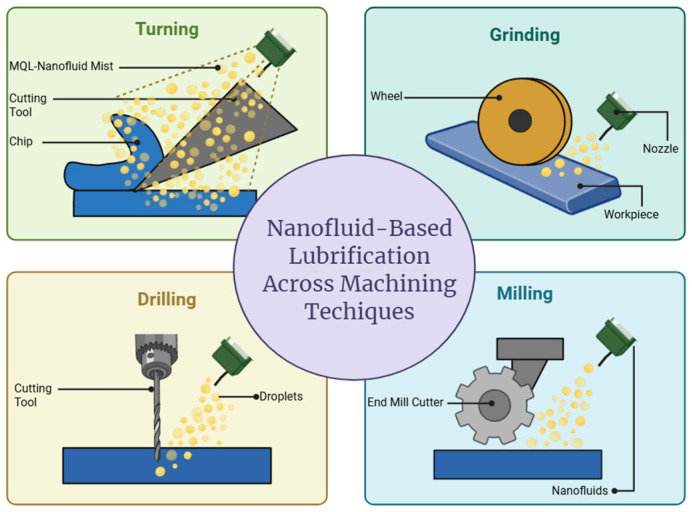
Schematic representation of nanofluid-based MQL systems applied to key machining operations: turning, milling, drilling, and grinding (created in Biorender.com).

There is vast evidence for the benefits of NFs in a variety of machining processes [313]. Figure 10 schematically illustrates NF-assisted MQL systems in turning, milling, drilling, and grinding, illustrating improved cooling, lubrication, and surface finish in all processes.

### 6.1. Machining Applications in Conventional Nanofluids

Conventional NFs have been shown unequivocally to outperform in various machining processes. In turning, one of the most investigated processes to utilize NFs, tool life, heat management, and surface integrity have been observed to improve significantly. Roy et al. [314] reported that incorporation of 3 vol.% Al_2_O_3_ with 1 vol.% multi-walled carbon nanotubes (MWCNTs) into the cutting fluid reduced cutting forces and energy consumption during high-speed turning of AISI 4140. MWCNTs contributed to lowering tool wear and localized temperatures. Similarly, Sharma et al. [315] also found that using Al_2_O_3_ with graphene nanoplatelets enhanced tribological performance during machining of AISI 304 by increased lubrication and reduced friction.

In milling, NF-enhanced MQL eliminates such problems as intermittent tool contact and cycle temperature load. Rahmati et al. [316] achieved improved surface quality by a MoS_2_-based nanolubrication system under pressurized air. Jamil et al. [317] showed that hybrid Al_2_O_3_-MWCNT NF enhanced the tool wear resistance and minimized energy consumption in milling of the notoriously hard-to-machine Ti-6Al-4V alloy.

Drilling, with its limited geometries and high thermal loading, is significantly improved by NF application. NFs assist in controlling surface pressure, enhance lubrication, and restrict contamination [318]. Huang et al. [319] employed a 2 wt.% NF-based MQL system during micro-drilling and attained lower drilling force and torque, along with improved hole quality.

In grinding, a process with high energy input and precise nature, NFs play an important role in improving performance. Lee et al. [320] utilized nanodiamond (ND)-filled NFs in MQL grinding and observed 33.2% decrease in normal force, 30.3% reduction in tangential force, and 64% decrease in surface roughness, attributed to the excellent thermal conductivity and hardness of NDs. In general, NFs improve force reduction during cutting, by 50% with Al_2_O_3_-based liquids [321], through improved lubrication and thermal conductivity. They also decrease tool wear forms such as crater, notch, and flank wear by lowering cutting zone temperature. Zhou et al. [322] achieved a 63% decrease in rake face crack wear by Fe_3_O_4_ in regular coolants, while Vázquez et al. [323] achieved a remarkable 604% enhancement in tool life using CuO NPs in mineral oil.

Overall, conventional NFs formulated using mineral or synthetic lubricants exhibit substantial advantages in turning, milling, drilling, and grinding. They extend tool life, reduce wear and frictional heat, and enhance machining efficiency, ultimately lowering operating costs and tool replacement rate.

### 6.2. Machining Applications in Green Nanofluids

The growing demand for sustainable, high-efficiency manufacturing has intensified scrutiny of conventional machining fluids, particularly due to their environmental and health impacts. Traditional petroleum-based cutting fluids, despite how important they are to lubrication, cooling, and chip evacuation, are associated with toxicity, poor biodegradability, work environment hazards, and costly disposal. Green NFs, biodegradable oils with NPs as additives and typically vegetable oil sources, have since emerged as environment-friendly alternatives. Notably, in the machining literature, the term green NF is more commonly used to refer to the natural source of the base fluid, rather than the synthesis approach of NP [324]. Unlike conventional base oils, vegetable oils such as coconut, canola, and soybean oils exhibit intrinsic advantages such as high viscosity indices, superior lubricity, and a natural capacity for film formation under extreme pressure conditions [325]. When combined with NPs like Al_2_O_3_, MoS_2_, graphene, or MWCNTs, the resulting colloids present synergistic effects that significantly improve machining outcomes, such as reduced cutting force and temperature, lower tool wear, enhanced surface finish, and decreased energy consumption [326]. Moreover, green NFs can also be compatible with MQL, enabling reduced fluid consumption as well as a better working environment due to negligible aerosol exposure [327]. Although turning is the best-studied green NF operation, encouraging results have been seen for milling and grinding too, with drilling still untouched.

In turning, constant tool–workpiece contact generates much heat, accelerating tool wear and degrading surface finish unless controlled correctly [328]. Investigations of the application of green NFs in MQL systems, typically colloidal suspensions of NPs in soybean oil, canola oil, or coconut oil, exhibit aggressive performance. For instance, MoS_2_-based NFs in soybean oil reduced surface roughness and flank wear in turning 90CrSi hardened steel [329]. Tool wear decreases of 34% were demonstrated by Hu et al. [330] with MWCNTs and 47.7% with MoS_2_, along with up to 51.8% surface roughness improvements at optimized conditions. Rahman et al. [331] demonstrated a considerable increase in roughness with Al_2_O_3_ in canola oil for Ti-6Al-4V ELI, while Rao et al. [332] found coconut oil-based NFs to improve chip formation, reduce friction, and improve dimensional accuracy. Other turning studies on AISI 1040 reported surface roughness decreases of 8.72% with the use of Al_2_O_3_-CNT in vegetable oil [332] and 47.8% with Al_2_O_3_ in vegetable oil alone [333]. Uses of nano-MoS_2_ in coconut oil reduced cutting forces by 37% [334]. Minh et al. [335] achieved the life of the tool improvement by 177% with Al_2_O_3_ in soybean oil. Padmini et al. [336] showed that MoS_2_ NFs in coconut oil (0.5 wt.%) reduced cutting forces by 37%, temperature by 21%, tool wear by 44%, and surface roughness by 39%, in comparison to dry machining. Additionally, hybrid NFs showed synergy in lubrication and cooling improvement, which suggests that NP type, concentration, and base oil can be optimized to provide better performance for specific machining conditions [337].

Even though they are advantageous, green NFs result in a few problems, such as stability of NP suspension, high-temperature oxidation of the bio-oils, short-circuiting, and even assembly uniformity in the cutting zone. Additionally, life-cycle assessments and toxicity tests must also be undertaken to validate their broader assertions of sustainability [338].

Under milling operations, the periodic cutting and fluctuating thermal loads can affect surface integrity and tool life [339]. Traditional flood cooling, although effective, has environmental issues with large fluid usage and disposal issues [340]. The employments of green NFs in MQL devices experience enhanced thermal management, lubrication, and reduced fluid usage [341]. Studies show that Al_2_O_3_–palm oil NFs with MQL improved thermal conductivity, reduced cutting forces, and surface finish during milling of Inconel 690 [340,342]. Hybrid NFs (e.g., MoS_2_ + graphite) have synergistic benefits, offering improved cooling and lubrication, extending tool life [343]. A water–olive oil nanoemulsion applied in machining AL6061 gave comparable chip temperature decrease and tool wear to business fluids with slightly poorer surface finish, illustrating effectiveness and sustainability [336]. Rahman et al. [344] contrasted tool wear through SEM among dry, traditional, and NF-MQL conditions. NFs significantly reduced build-up edge formation and tool wear by the thermal conductivity and lubricity of suspended NPs. Despite encouraging developments, few major challenges to green NF application for milling still exist. They are stability of NP suspension, vegetable oils’ resistance to oxidation at elevated temperatures, and efficient delivery in MQL systems. Furthermore, long-term effects on machine components and environment safety need to be assessed to make it industrially viable [345,346].

Drilling, a key machining operation, is difficult due to restrained cutting zones, chip evacuation problems, and vast amounts of heat generated, with the possibility of enhancing tool wear and reducing dimension accuracy. Conventional flood cooling cannot properly address these issues and is also health- and environment-damaging. The effectiveness of green NFs in MQL-assisted drilling is uncovered by recent studies [346]. For instance, nanodiamond green NFs optimized through response surface methodology and genetic algorithms exhibited reduced thrust force and torque and enhanced material removal rate [347]. Eltaggaz et al. [348] achieved 26.5% flank wear reduction when they used Al_2_O_3_ in vegetable oil. Pal et al. [349] employed 1.5 wt.% Al_2_O_3_ in vegetable oil for drilling AISI 321 stainless steel at reduced thrust forces, lower torque, better surface finish, and lesser tool wear as compared to dry, flood, or conventional MQL conditions. Mosleh et al. [350] used MoS_2_ and hBN NPs in MQL aerosols in titanium orbital drilling and achieved improved lubrication, less tool transfer film, and consistent friction behavior in the tool-workpiece interface.

**Figure 11:**
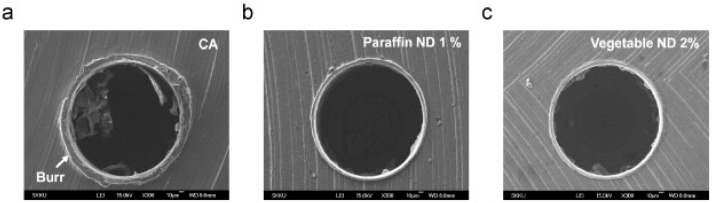
SEM micrographs of the drilled holes in Al601 under different lubrication conditions: (**a**) compressed air lubrication (Run 1), (**b**) NF-MQL with paraffin oil + 1 vol.% of nanodiamond (Run 4), and (**c**) NF-MQL with vegetable oil + 2 vol.% of nanodiamond (Run 7). Reproduced from [351].

A comparative study by Nam et al. [351], seven lubrication conditions for micro-drilling Aluminum 6061 using uncoated carbide twist drills under MQL with various base fluids (paraffin vs. vegetable oils) and concentrations of nanodiamond (1 and 2 vol.%). Results showed NF-MQL significantly outperformed pure MQL and compressed air. The best performance was obtained with 1 vol.% nanodiamond in paraffin oil, reducing torque by 31.3% and thrust force by 32.2% when compared to compressed air. For vegetable oil-based lubricants, 2 vol.% nanodiamond showed similar enhancements (31.3% reduction in torque, 30.9% reduction in thrust force). These trends were in agreement with the higher viscosity of vegetable oils, requiring higher NP concentrations in order to attain efficient performance. SEM micrographs (Figure 11) revealed improved chip evacuation and burr removal under optimum NF-MQL conditions for both lubricants.

Grinding is an energy-intensive operation where excessive temperatures are sufficient to compromise surface integrity and dimensional tolerances. While traditional flood cooling facilitates heat removal, its application of mineral oil-based fluids is environmentally and health hazardous. Like other machining processes, green NFs in MQL systems offer a more environment-friendly alternative, providing effective lubrication and thermal management with reduced fluid consumption [352,353]. While grinding Ti-6Al-4V, Zhang et al. [354] reported the lowest specific grinding energy (51.96 J/mm^3^) and friction coefficient (0.60) when cryogenic air was added to NF-MQL in comparison to both separate processes—because of better film stability and atomization of droplets. Palm oil NF decorated with carbon nanotubes, in another study, achieved the lowest grinding temperature (110.7 °C) and highest convective heat transfer coefficient (1.3 × 10^4^ W/m·K) during grinding of a nickel alloy [355]. In addition, Al_2_O_3_ NPs-based NFs in cottonseed oil for MQL milling of 45 steel showed that 0.5 wt.% gave the best performance: minimum specific energy (114 J/mm^3^), surface roughness (1.63 μm), and best wettability, showing the synergistic benefits of NPs and eco-friendly base fluids [356].

A few severe challenges of green NF application in grinding are stability of NP suspensions, resistance to oxidation of vegetable oils, and optimization of delivery systems in MQL systems [326,357].

### 6.3. Comparative Insights into Green and Conventional Derived Nanofluids for Machining Applications

The environmental impact of conventional cutting fluids is significant during both use and disposal. NFs, especially when used with MQL systems, greatly reduce fluid volume, waste, and associated costs. Vegetable oil-based NFs offer biodegradable, less hazardous alternatives to mineral oils, supporting sustainable manufacturing. Although their initial production cost is slightly higher, benefits like longer tool life, reduced energy use, and improved surface quality lead to overall cost savings. In machining processes NFs are more effective due to their higher thermal and tribological performance. This results in better surface finish, less tool wear, lower cutting forces, and enhanced tool life. Also, with the integration of NFs and MQL, environmental damage and operation costs are reduced, which concurs with the strengthening call for sustainable and high-performance machining. Experiments show that both conventional and green NFs significantly improve machining performance, with green NFs sometimes even outperforming conventional ones. This may be due to triglycerides present in vegetable oils that result in improved lubrication and heat transfer [337]. Green NFs also provide environmentally friendly advantages, breaking down environmentally benign compounds through enzymatic or chemical processes and do not require filtration prior to disposal, which facilitates their disposal in an eco-friendly manner [324].

Table 3 compares performance for machining operations, NP types, sizes, and base fluids. The two NF types perform better on key metrics, but green NFs perform better on surface quality and tool wear when optimized for concentration and delivery. However, it is important to note that most studies evaluate either conventional or green NFs formulations independently, which restricts the ability to generalize conclusions and identify clear performance hierarchies. Direct, side-by-side comparisons between both types under equivalent experimental conditions are rare. This highlights a clear gap in the literature and underscores the need for more systematic investigations directly contrasting conventional and green NFs performance under controlled conditions. Moreover, while the sustainability of green NFs is often assumed, comprehensive life-cycle assessments and toxicity studies are rarely included in the analysis. Without full environmental and economic audits, spanning NP production, base oil cultivation, fluid formulation, and disposal, the true sustainability of these fluids remains only partially supported.

## 7. Costs and Industrial Commercialization of Nanofluids

NFs have been widely studied for applications in fields such as renewable energy systems, automotive radiators, electronic cooling, and industrial heat exchangers. Despite the extensive scientific research and proven laboratory-scale performance improvements, the transition from research to real-world industrial applications has been slow, with only a few successfully implemented. Some key causes for such unsuccessful industrial implementation include long-term stability, high production and operational costs, inconsistency in reported thermophysical properties of NFs, and higher pumping power compared to conventional working fluids [363,364].

### 7.1. Economic Considerations in Nanofluid Production and Performance

The economic viability of NF technology is largely dependent on production cost, which is typified by a dominance of (90–95%) NP synthesis [363]. Karthikeyan et al. [365] set the commercially available NPs such as Al_2_O_3_ (20 nm), TiO_2_ (50 nm), CuO (20 nm), MWCNTs (20 nm), and Cu (40–60 nm) from USD 225/kg (Al_2_O_3_) to USD 1560/kg (Cu), with significant impact on costs of producing NFs. Further, NF unit cost also responds to NP size and concentration (e.g., SiO_2_- and Ag-based NFs at 0.04% concentration cost EUR 1186/m^3^ and EUR 223,473.06/m^3^, respectively) [364,366]. For such a high price tag, researchers like Charitidis et al. [367] argue that conventional NP synthesis is economically not feasible for commercial application. Alternatives such as green synthesis or NP recovery from waste by industries are viable cost savers [277,278]. Economic analysis by Karthikeyan et al. [365] between MWCNT–CuO NFs and water in a heat exchanger showed that only one of the four cases with NF stability assumed for 5 years was economical, with a payback period of below 1 year. In all other cases, instability-based periodic costs and replacement of the fluid rendered NFs uneconomical. There are also evaluations based on other indices like the price performance factor [368], efficiency-price index [369], and price performance index [363,370]. All these are founded on factors like thermal conductivity, pumping power, flow regime, and NP cost [363,370].

Even as insightful, the studies above are not on a large enough scale. Larger-scale examination of different NF formulations, operating conditions, and system types is required to validate economic models and ensure real-world applicability.

### 7.2. Commercialization Attempts and Barriers to Industrial Implementation

Although NFs have superior thermophysical properties compared to conventional fluids, commercialization is negligible at present. Some attempts (e.g., by Hydromx, NanoHex, and Nano Research Lab) have been made to mass produce NF for industrial use [363,371]. Hydromx, for example, produced a glycol-based NF that saved energy by 20–35% and recovered ROI within three years in well over hundreds of installations [372].

However, mass commercialization is hindered with enormous challenges. High production and stability remain basic issues. NPs are susceptible to Van der Waals and gravitation force-driven agglomeration, causing sedimentation that worsens thermal performance and has the potential to clog, especially in microchannel systems [364,373]. Moreover, non-uniform measurements of thermal properties create scientific doubt. This was demonstrated by Buongiorno et al. [374], who distributed the same NF samples to several labs and reported up to 10% thermal conductivity variation depending on procedures, a result of a lack of standardized synthesis and testing protocols [363,375]. Another problem is corrosion and equipment erosion risk in industry. For instance, Al_2_O_3_ NFs enhanced carbon steel erosion and corrosion rates by 237% compared to seawater [364,376] and thereby raises questions regarding long-term system durability.

### 7.3. Environmental Implications and Sustainability

The life cycle of NFs has the entire environmental impact—from energy-intensive synthesis of NP material to waste handling and recycling problems. NPs like Ag, Cu, and Zn contain metals with toxicity and bioaccumulation problems, but comprehensive life-cycle analysis (LCA) is scarce [363]. Life-cycle research on biodegradable nanomaterials (e.g., copper ferrite NFs based on *Chlorella vulgaris*) and environmentally friendly synthesis routes is forthcoming but in the nascent stages with no commercial scalability yet [278,363]. Sustainability applications focus on applications where NFs are employed to enhance energy efficiency of heat exchangers [377], enhance thermal system recovery [378], and reduce cutting fluid usage in manufacturing [379,380]. There is limited reusability due to NP aggregation, sedimentation, and chemical degradation. Surfactants [381], sonication [382], and magnetic separation [383] are solutions used in an attempt to recover, with recovery techniques such as ultracentrifugation, electrophoresis, and filtration also being considered [363].

Even with advances, few studies have investigated the full environmental trade-offs of NFs. Differences in toxicity between individual NPs and final products are rarely addressed [384]. For more complete understanding and reduction of these impacts, future work must adopt consistent, lifecycle-based sustainability evaluations.

## 8. Conclusions

This review has highlighted the remarkable potential of NFs across diverse domains, including machining, solar energy, and biomedical applications, where their superior thermophysical and multifunctional properties contribute to improving performance and efficiency. In machining processes, the use of NFs improves surface quality, extends tool lifespan, and reduces operating costs. In heat exchanger systems, they offer improved heat transfer and optical absorption, thereby increasing the overall energy efficiency and enabling a reduction in equipment size. In the biomedical field, NFs facilitate advances in diagnostics, targeted drug delivery, and photothermal therapies, particularly with magnetic and metallic NPs.

Despite these significant advances, key challenges continue to delay the industrial and clinical adoption of NFs. Issues such as long-term stability, increased viscosity at higher concentrations, high production costs, and the absence of standardized synthesis and regulatory protocols must be addressed. While strategies such as surface modification, the application of external magnetic fields, and green synthesis approaches have shown promise in mitigating some of these limitations, further research is required.

Moving forward, research should aim to optimize NP characteristics, such as size, shape, and concentration, through standardized experimental methodologies, improved predictive models, and prolonged durability tests. In parallel, scaling up green synthesis methods demands greater control over critical parameters, including pH, temperature, and the concentration of bioreducing agents, to ensure reproducibility and industrial feasibility. The application of NPs produced by green synthesis in the field of machining remains an unexplored area of knowledge, with great potential compared to conventional NFs, given the observed improvements in thermal properties and stability. However, it is also necessary that new studies comparing the properties of NFs produced by conventional methods and green synthesis be carried out following equivalent procedures, since such studies are scarce in the biomedical field and almost nonexistent in the area of heat transfer. These studies will allow the potential advantages identified in NPs produced by green synthesis to be assessed more objectively. Moreover, there is a pressing need to establish regulatory and standardization frameworks that define protocols for assessing stability, toxicity, and environmental impact. Such efforts would benefit from close collaboration among material researchers, toxicologists, and regulatory authorities, ensuring that NF technologies will meet safety and efficacy criteria for both industrial and biomedical uses.

By addressing these research gaps, particularly the long-term stability, material optimization, scalable green synthesis, and regulatory alignment, this field can finally move towards the commercialization of NFs. This would enable the development of next-generation thermal systems and medical technologies that are not only high-performing and cost-effective but also environmentally sustainable and safe for widespread use.

## Figures and Tables

**Table 1:** Comparative summary of green and chemically synthesized nanofluids for biomedical applications evaluated under similar experimental conditions.

NP	Synthesis Approach	Toxicity and Biocompatibility	Environmental Impact	Main Differences	Applications	Ref.
**Ag**	Chemical: Sodium citrate reduction	High toxicity to human dermal fibroblasts (HDFa); ROS ↑; non-selective cytotoxicity	Chemical waste, higher energy input	Higher cytotoxicity, chemical residues, less biocompatibility	Antibacterial, basic biomedical	[210]
	Green: *Azadirachta indica* (Neem) leaf extract	Non-toxic to HDFa and RBCs; selective cancer cell (NCI-H460) apoptosis; ROS ↑ only in cancer cells	Eco-friendly, minimal waste	Safer profile, selective cancer cytotoxicity, natural capping/stabilizing agents	Anticancer, biomedical, drug delivery	
**Ag**	Chemical: Sodium citrate method	Reduced antioxidant activity; lower DPPH scavenging %	Lower stability, generates waste	Weaker antioxidant capacity, poor stability over time	General lab use, short-term antimicrobial	[211]
	Green: *Mussaenda frondosa* leaf extract	Higher antioxidant activity (↑ DPPH scavenging); non-toxic profile	Sustainable, minimal chemical load	Better stability, phytochemical surface functionalization	Biomedical, antioxidant applications	
**Ag**	Chemical: Commercial (Sigma-Aldrich)	MIC = 8 µg/mL (vs. *S. aureus*); lower biofilm inhibition	Synthetic chemicals; not eco-friendly	Less effective at lower doses, weaker biofilm suppression, stable colloid	Antibacterial, anti-biofilm	[214]
	Green: *Zataria multiflora* extract	MIC = 4 µg/mL (vs. *S. aureus*); better biofilm inhibition at 0.5–2× MIC	Eco-friendly; plant-based	Better inhibition at low conc., phytochemical capping, stable at pH 9	Antibacterial, anti-biofilm	
**MgO**	Chemical: NaOH + Magnesium acetate	Smaller inhibition zones against *B. subtilis*, *E. coli*, etc.	Requires strong base; more energy input	Larger particles, lower activity	General antimicrobial	[212]
	Green: *Lawsonia inermis* extract	Larger inhibition zones at all tested doses (20–80 µL)	Green route, plant-derived	Better porosity, biocompatibility	Biomedical, antimicrobial	
**TiO_2_**	Chemical: Hydrothermal	Lower antibacterial effect	Ethanol use; higher temp calcination	Lower photocatalytic and antimicrobial efficiency	Photocatalysis, bactericide	[213]
	Green: *Jasmine flower* extract	Higher inhibition zones	Minimal by-products, bio-safe	Higher biological activity, cleaner synthesis	Photocatalysis, antibacterial	

**Table 2:** Comparative summary of green and chemically synthesized nanofluids for heat transfer applications.

NP	Synthesis Approach	Base Fluid	Thermal Conductivity Increase	Colloidal Stability	Types of Testing	Ref.
Ag	Microwave- assisted chemical precipitation	Distilled water	Up to ~29% at 1 vol.%	----	Thermal conductivity and viscosity measurements in static fluid (KD2 Pro, Brookfield viscometer)	[294]
	Green tea (*Camellia sinensis*)	Water–Ethylene Glycol 50%	Up to 37% at 1 vol.%	Up to 12 days	Forced convection test in a double-pipe heat exchanger (flowing fluid); thermal conductivity measured with KD2 Decagon	[289]
MWCNT	Conventional two-step with gum Arabic (0.25 wt.%) and 3 h ultrasonication	Deionized water	Up to 23% at 0.8 vol.%	Stable (confirmed by UV spectroscopy)	Thermal conductivity (KD2 Pro), specific heat (DSC), in static fluid	[295]
MWCNT	Green: Clove (*Syzygium aromaticum*)	Deionized wate	Up to 20% at 0.08 wt.%	Up to 60 days	Thermal conductivity and viscosity measurements in static fluid	[290]
GNP	Direct dispersion in distilled water via ultrasonication	Distilled water	Up to ~31% at 0.1 wt.%	------	KD2 Pro thermal analyzer	[296]
CGNP	Green: Clove (*Syzygium aromaticum*)	Deionized water	Up to 22.92% at 0.1 wt.%	Up to 63 days (UV–Vis analysis)	Thermal conductivity (KD2 Pro) in static fluid	[291]
GGNP	Green: Gallic acid	Distilled water	Up to 17.76% at 0.1 wt.%	>60 days without agglomeration (confirmed by visual inspection and stability curve)	Thermal conductivity via KD2 Pro analyzer; application in flat-plate solar collector (forced flow system, 0.5–1.5 L/min)	[283]
SiO_2_	Conventional Stöber method	Ethanol	Up to ~60% at 1.17 vol.%,	------	KD2 Pro—Transient Hot-Wire	[297]
SiO_2_	Green: Rice husk	Deionized water	Up to 38.2% at 3.0 vol.%	>180 days	Thermal conductivity: transient hot-wire (KD2 Pro)	[282]
CuO	Two-step method	Etilenoglicol –Water (40:60)	36.97% at 2.0 (wt.%)	75 days	KD2 Pro Transient hot-wire	[298]
CuO	Green: Callistemon viminalis	Deionized Water	Up to 34% at 9% (vol.%)	Stable with PVP	KD2 Pro Transient Hot-Wire	[292]

**Table 3:** Performance evaluation of conventional (Conv.) vs. green NFs throughout various machining applications.

Types of NPs	Size of NPs (nm)	Approach	Base Fluid	Process	Performance	Ref.
MoS_2_	<5	Conv.	Castrol Syntilo 9930	Turning	Lowered cutting and feed forces, cutting zone temperature, tool wear, and improved surface finish compared to the conventional coolant.	[358]
		Green	Waste coconut oil			
Nano boric acid	50	Conv.	SAE-40 oil		Suspensions at 0.5% concentration significantly reduced cutting temperature, flank wear, and surface roughness, with coconut oil showing superior performance over SAE-40 due to better lubricating properties.	[359]
		Green	Coconut oil			
Al_2_O_3_	20	Conv.	Traditional cutting fluid	Milling	At 1.0 vol.% and 20 nm, the palm oil-based Al_2_O_3_ NF reduced workpiece surface temperature by more than 10 °C and decreased milling force deviations by 8–13% compared to conventional cutting fluid.	[360]
		Green	Palm oil			
MoS_2_	50	Conv.	Liquid paraffin 2 wt.%	Grinding	In MQL grinding of Grade 45 steel, the MoS_2_–soybean oil NF significantly lowered grinding forces and friction while improving G-ratio and surface quality compared to a comparable MoS_2_–paraffin oil NF under identical conditions.	[361]
		Green	Palm oil 2 wt.%			
			Rapeseed oil 2 wt.%			
			Soybean oil 2 wt.%			
MoS_2_	~250	Conv.	Cimtech500		Reduction in grinding forces (up to 27%) and enhanced G-ratio (up to 46%), with soybean oil achieving a 9% force reduction and 15% G-ratio improvement compared to its base oil alone, with minimal fluid usage (5 mL/min).	[362]
			Paraffin oil			
		Green	Soybean oil			
			CANMIST oil			
ND	30	Conv.	Paraffin oil	Drilling	1 vol.% achieved the greatest reduction in torque (31.3%) and thrust force (32.2%), while 2 vol.% led to a slight performance decline due to possible particle agglomeration.	[351]
		Green	Vegetable oil		At 2 vol.%, the NF matched paraffin oil in reducing torque (31.3%) and thrust force (30.9%), while significantly improving chip evacuation and burr removal.	
